# A Comprehensive Review of Autophagy and Its Various Roles in Infectious, Non-Infectious, and Lifestyle Diseases: Current Knowledge and Prospects for Disease Prevention, Novel Drug Design, and Therapy

**DOI:** 10.3390/cells8070674

**Published:** 2019-07-03

**Authors:** Rekha Khandia, Maryam Dadar, Ashok Munjal, Kuldeep Dhama, Kumaragurubaran Karthik, Ruchi Tiwari, Mohd. Iqbal Yatoo, Hafiz M.N. Iqbal, Karam Pal Singh, Sunil K. Joshi, Wanpen Chaicumpa

**Affiliations:** 1 Department of Genetics, Barkatullah University, Bhopal 462 026, Madhya Pradesh, India; 2 Razi Vaccine and Serum Research Institute, Agricultural Research, Education and Extension Organization (AREEO), Karaj 31975/148, Iran; 3 Division of Pathology, ICAR-Indian Veterinary Research Institute, Izatnagar, Bareilly 243 122, Uttar Pradesh, India; 4 Central University Laboratory, Tamil Nadu Veterinary and Animal Sciences University, Madhavaram Milk Colony, Chennai, Tamil Nadu 600051, India; 5 Department of Veterinary Microbiology and Immunology, College of Veterinary Sciences, UP Pandit Deen Dayal Upadhayay Pashu Chikitsa Vigyan Vishwavidyalay Evum Go-Anusandhan Sansthan (DUVASU), Mathura, Uttar Pradesh 281 001, India; 6 Sher-E-Kashmir University of Agricultural Sciences and Technology of Kashmir, Shalimar, Srinagar 190025, Jammu and Kashmir, India; 7 Tecnologico de Monterrey, School of Engineering and Sciences, Campus Monterrey, Ave. Eugenio Garza Sada 2501, Monterrey, N. L., CP 64849, Mexico; 8 Department of Pediatrics, Division of Hematology, Oncology and Bone Marrow Transplantation, University of Miami School of Medicine, Miami, FL 33136, USA; 9 Center of Research Excellence on Therapeutic Proteins and Antibody Engineering, Department of Parasitology, Faculty of Medicine Siriraj Hospital, Mahidol University, Bangkok 10700, Thailand

**Keywords:** autophagy mechanism, autophagy-associated diseases, macroautophagy, chaperone-mediated autophagy, apoptosis, necroptosis, necrosis, iron homeostasis, autophagy inhibition, AKT/mTOR signaling pathway, autosis

## Abstract

Autophagy (self-eating) is a conserved cellular degradation process that plays important roles in maintaining homeostasis and preventing nutritional, metabolic, and infection-mediated stresses. Autophagy dysfunction can have various pathological consequences, including tumor progression, pathogen hyper-virulence, and neurodegeneration. This review describes the mechanisms of autophagy and its associations with other cell death mechanisms, including apoptosis, necrosis, necroptosis, and autosis. Autophagy has both positive and negative roles in infection, cancer, neural development, metabolism, cardiovascular health, immunity, and iron homeostasis. Genetic defects in autophagy can have pathological consequences, such as static childhood encephalopathy with neurodegeneration in adulthood, Crohn’s disease, hereditary spastic paraparesis, Danon disease, X-linked myopathy with excessive autophagy, and sporadic inclusion body myositis. Further studies on the process of autophagy in different microbial infections could help to design and develop novel therapeutic strategies against important pathogenic microbes. This review on the progress and prospects of autophagy research describes various activators and suppressors, which could be used to design novel intervention strategies against numerous diseases and develop therapeutic drugs to protect human and animal health.

## 1. Introduction

Autophagy is a conserved catabolic process that is involved in cellular homeostasis and is required to maintain normal cellular physiology under stressful conditions [1]. It overcomes carcinogenic, infectious, degenerative, and deleterious agents to maintain the homeostasis of bodily systems and regulate healthy life processes; thus, its dysregulation is known to cause multiple human diseases [2,3,4,5]. Autophagy can be a selective or non-selective lysosomal degradative process and is activated by stresses such as starvation or rapamycin via regulatory signaling complexes [6,7]. There are three types of autophagy: macroautophagy, microautophagy, and chaperone-mediated autophagy (CMA) [8]. Macroautophagy, referred to as “autophagy” from now on, is the major pathway which engulfs large portions of cytoplasm and cellular contents (e.g., long-lived proteins, aggregated proteins, damaged organelles, and intracellular pathogens) into a double-membraned vacuole called the autophagosome, which fuses with lysosomes to form an autolysosome, degrades the autolysosomal contents, and recycles macromolecules for reuse [9,10,11]. 

Microautophagy refers to the process by which lysosomes directly engulf and digest small volumes of cytosolic substrate [12,13], whilst CMA is induced by physiological stresses such as prolonged starvation [14] and involves the heat shock cognate protein (HSC70; 71-kDa, also known as HSPA8) which contains a KFERQ-like pentapeptide sequence [15]. The CMA pathway delivers target proteins across lysosomal membranes into the lysosomal lumen by interacting with lysosome-associated membrane protein type 2A (LAMP-2A) [9]. Hence, CMA differs from microautophagy and macroautophagy, as it does not require vesicular trafficking [14]. Regardless of the type, autophagy acts as a cleaning mechanism by removing or degrading unnecessary materials from the body (e.g., proteins, organelles, and microbes) and retaining or maintaining materials (biochemicals, metabolites, and organelles) required for survival, function, and development [1,6,16,17]. The physiological processes of autophagy are governed by numerous cellular regulators (e.g., transcription factors and genes), which can affect homeostatic processing if disturbed by genetic or functional reasons, or overexertion [4,6,17]. Hence, autophagy defects can affect the pathogenesis of many diseases [17]. 

The roles of autophagy have been explored in fields such as health, disease, infection, degeneration, and genetic or lifestyle-acquired diseases [18,19,20,21]; however, cancer [7,22], microbial infections [20,21,23], and degenerative diseases [18,24] have been the main focus of autophagy-related research. Currently, the roles of autophagy are being explored in diverse fields of study.

Autophagy plays important roles in cancer metastasis; 4-acetyl-antroquinonol B has been shown to modulate autophagy and prevent the growth of ovarian cancer cells [25]. Clinical studies have revealed higher levels of autophagic flux in distant metastases than in primary tumors [26,27]; therefore, autophagy has stage-dependent dual roles in cancer which may facilitate the growth and spread of tumors and affect treatment resistance [19]. Conversely, autophagy has been shown to act as a tumor suppressor during the early and late phases of cancer development [28,29] by mediating the destruction and removal of carcinogens and cancerous cells, thus enabling the growth and development of healthy cells; however, under disturbed or uncontrolled conditions autophagy can promote cancer growth and dissemination. Autophagy is also important in neuronal homeostasis, with its dysfunction associated with numerous neurodegenerative disorders [18]. Pathogenic protein aggregates are a common feature of neurodegenerative disorders, and dysfunctional autophagy is involved in this disease state [30]. Furthermore, mutations in autophagy regulation genes have been shown to induce neurodegenerative diseases such as amyotrophic lateral sclerosis, Alzheimer’s disease, and familial Parkinson’s disease [18,24]. These mutations affect different stages of autophagy and thus have different implications for pathogenesis and therapy [31].

In the modern world, factors such as globalization, liberal trade, climate change, population explosions, public health lapses, immune pressures, and mutations, species jumping, and emerging antibiotic resistance in pathogens have facilitated the spread of various infectious pathogens. In recent years, it has been shown that autophagy has a role in many emerging and re-emerging infectious viral and bacterial diseases that pose significant threats to humans. Autophagy initially encounters these infectious pathogens to neutralize them; however, may infections can propagate themselves as persistent intracellular infections and are generally associated with wide outbreaks, epidemics, and highly devastating effects. Many viral life cycles are linked with autophagic pathways. The influenza A virus induces autophagosome formation during the early stages of infection and inhibits autophagosomal maturation during the later stages. Classical swine fever virus replication is negatively regulated by mTORC1 via autophagy and IRES-dependent translation, whilst Dengue/Zika virus pathogenicity is modulated by antibody-dependent enhancement (ADE) which can induce autophagy in human umbilical vein endothelial cells. Multimodal necrotic cell death is driven by open reading frame-3a of severe acute respiratory syndrome (SARS)-coronavirus (CoV), that triggers the lysosomal damage and dysfunction and therefore transcription of autophagy-related genes is enhanced, whilst endoplasmic reticulum (ER) stress in Dengue virus (DENV) infections results in autophagy activation, viral replication, and pathogenesis. The disruption of mitochondrial membrane potentials by the non-structural protein of Crimean-Congo hemorrhagic fever (CCHF) virus results in apoptosis, whereas paramyxovirus V proteins inhibit constitutively active MDA5 proteins to induce autophagy. All of these viral events are related to autophagy and can provide directions for future therapies for Chikungunya (CHIKV), DENV, and Zika virus (ZIKV) infections. Autophagy has a pivotal role in viral diseases such as bird flu [32], swine fever [33], Ebola virus disease [20], ZIKV infection [34,35], SARS [36], CHIKV infection [37], DENV infection [38], viral encephalitis [39], CCHF [40], Hendra virus (HeV) infection [41], Nipah virus (NiV) infection [42], and the West Nile virus (WNV) infection [43]. Apart from these, other viral diseases, such as rabies, rotavirus enteritis, and smallpox, have already posed a serious threat to human life [44,45,46,47,48]. Autophagy has also been shown to have a central role in microbial infections [49], including those caused by *Listeria* [50], *Salmonella* [51], *Shigella* [52], and *Streptococcus* [53]. Autophagy can kill or eradicate infectious disease-causing pathogens via the autophagosome or autophagolysosome (autolysosome) to prevent or treat infection [20,21]; however, autophagy can also disseminate pathogens during pathogenesis. For example, gut epithelial autophagy can disseminate viruses and bacteria in enteric diseases. Therefore, autophagy can play a dual role in infections [20,21,54]. 

In recent years, there has been an increase in the incidence of lifestyle and genetic diseases, such as cancers and neurodegenerative disorders (Alzheimer’s, Parkinson’s, and Huntington’s diseases), which affect the quality of life. Advances in science and technology have contributed to overcoming these challenges. Novel, alternative, and complementary therapeutic options have been developed, including phages, homing peptides, cytokines, siRNA, viral inhibitors, Toll-like receptors (TLRs), antibodies, probiotics, herbs, phytomedicines, nanomedicines, and immunomodulatory techniques [55,56,57,58,59,60,61,62,63,64]. Autophagy is the first mechanism to clear endogenous debris and exogenous substances and maintains normal physiological conditions in all eukaryotic cells [65]. Besides maintaining homeostasis [66], autophagy also regulates the development [67], differentiation [5], and maturation [68] of cells, such as endothelial cells [69], erythrocytes [70], and adipocytes [71,72]. These cells are involved in normal physiological (e.g., erythrocytes in respiration), immunological (e.g., mononuclear cells in immunity), metabolic (e.g., adipocytes in fat metabolism), growth (e.g., osteocytes in bone growth), and development (e.g., spermatozoa or ova in reproduction) processes. Autophagy is also involved in clearing abnormal protein accumulations and correcting mitochondrial disorganization [73,74]. The processes of autophagy and apoptosis are interwoven and have been implicated in both microbial infections [54,75] and cancers [26,76]. Autophagy might play both physiological and pathological roles since it is involved in overcoming cell stresses [19,77,78]. Considering the numerous roles and functions of autophagy in health and disease, we present a comprehensive overview of autophagy, its mechanisms and types, and its associations with other cell death mechanisms. The dual roles of autophagy in infectious diseases (bacterial and viral), tumor suppression/progression, brain development/neurodegeneration, the immune system, and autoimmune diseases, and its other roles have been discussed thoroughly alongside numerous applications of autophagy. We have also summarized the role of autophagy in cardiovascular diseases, iron homeostasis, obesity, diabetes, and diseases caused by defects in autophagy genes. The treatment of autophagy-associated diseases has been described alongside strategies to inhibit or activate autophagy in the prevention and treatment of diseases. This review details the important functions of autophagy in health and disease and its key roles in disease prevention and treatment.

## 2. Autophagy: A Brief Overview 

Autophagy (from the Greek words *auto*, meaning self, and *phagy*, meaning eating), is an essential, ubiquitous, evolutionarily conserved, catabolic, and self-degradative process that mediates the destruction of cytoplasmic macromolecules to preserve genomic integrity, achieve cell metabolism, and ensure cell survival [30,79,80,81]. It is a natural regulatory mechanism which retains beneficial substances and removes harmful substances from body, whilst playing a housekeeping role in the elimination of misfolded or aggregated proteins, the eradication of damaged organelles, proteins [82,83,84], and cancerous materials [7], and the elimination of foreign pathogens such as viruses via a degradative lysosomal pathway [21,85,86,87]. 

Numerous physiobiological roles of autophagy have been identified, such as the disposal of endogenous wastes and exogenous agents to maintain homeostasis; however, disturbing the natural balance of this mechanism can result in pathological consequences [88]. 

Since it is the primary system for cleaning the body, autophagy can prevent or treat cancer by killing cancerous cells and degrading endogenous or exogenous carcinogens; thus, favoring the development of healthy cells. However, autophagy may have dual roles in cancer as it is involved in stem cell-related resistance to anti-cancer therapy (radioresistance and chemoresistance), metastasis, and tumor recurrence [89]. As obligate intracellular pathogens, viruses interact with multiple host cell processes for their survival, including metabolism, cellular trafficking, and immunity-related responses [54,90]. Furthermore, autophagy is a major degradative cellular process, with essential roles in many innate and adaptive immune processes [91,92,93]. Autophagy also regulates the phosphorylation of p38 and ERK1/2 MAPKs in BV2 microglial cells, required for nitric oxide production [94,95]. Thus, it can affect the activation of neuronal cells by microglia and suppress neurotoxicity. Moreover, it can downregulate pro-inflammatory mediators in BV2 microglial cells to rescue them from LPS- and α-synuclein-induced neuronal cell death [94].

Autophagy can either be selective or non-selective [96]. In selective autophagy, cargo is recognized by specific receptors to enable their specific identification, sequestration, and degradation by the autophagosome, whereas in non-specific autophagy, all materials are degraded by the lysosome in a non-specific manner [96,97]. Furthermore, autophagy is known to exist in two forms: constitutive and reactive (induced) autophagy. Constitutive autophagy has not been well studied, whereas the latter has been studied extensively and is known to stimulate neurite remodeling in developing brains, thus may be essential during brain development [98,99]. Mice lacking the autophagy proteins Atg59 and Atg710 display excessive neurodegeneration, indicating that autophagy has physiological importance [100]. Numerous factors relating to nutrient deprivation (amino acids and hormones) and build-up of degraded products (proteins) or exogenous agents (pathogens) have been evaluated as induced autophagy stimuli [101,102]. Endogenous and exogenous stimuli induce autophagy for degradation or as a repair mechanism.

Several stimuli have been shown to induce autophagy, including stress, amino acid starvation [103], rapid declines in trophic factors or hormones (such as sex-based differences) [104], lipid starvation [105], impaired intracellular cholesterol trafficking [106], protein products, and infectious pathogens [32,38,85]. These stimuli can affect the autophagic function and induce different morphological consequences via diverse signaling pathways; for instance, suppressing phosphatidylinositol-3-kinase (PI3K) inhibitors and Beclin 1 inhibits the starvation-induced mitochondrial autophagy, but not the neurotoxin (1-methyl-4-phenylpyridinium)-mediated autophagy [107,108,109]. Although autophagy was discovered over 50 years ago [54], its molecular mechanisms were only understood in the late 1990s following a genetic screening in yeast, which revealed mutations in autophagy-related genes. At least 30 yeast autophagy genes (*Atgs*) have been identified, many of which have mammalian cell homologs [85].

Many molecular mechanisms have been explored to reveal the basic processes underlying autophagy. Multiple signaling pathways focus on two protein complexes to initiate autophagy, the ULK1 (unc51-like autophagy activating kinase 1) protein kinase complex and the PI3KC3-C1 (class III phosphatidylinositol 3-kinase complex I) lipid kinase complex [110]. Novel autophagy regulators with RNA-related activities have also been shown to be involved in this process [111]. Furthermore, upstream signaling pathways common to both autophagy and apoptosis are known to be induced by ER stress via signaling molecules such as PERK/ATF4, IRE1α, ATF6, and Ca^2+^ [112]. The details of these mechanisms will shed light on the different forms of autophagy and the numerous intermediates involved. 

**Figure 1:**
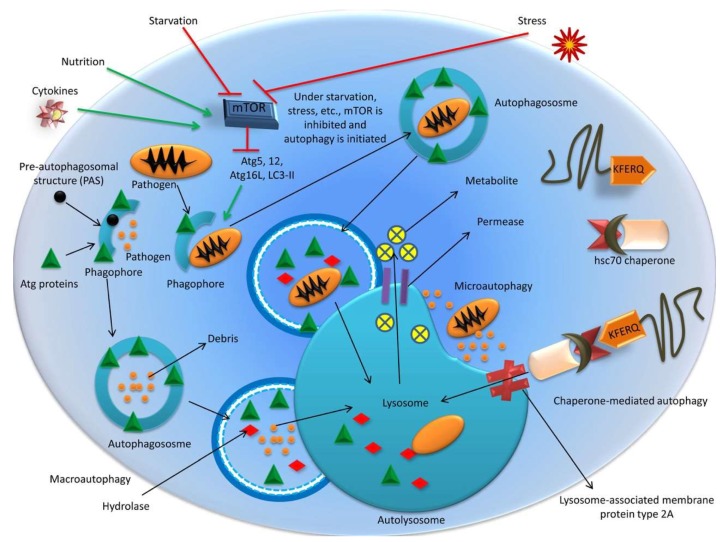
Different types of autophagy. Macroautophagy, microautophagy, and chaperone-mediated autophagy.

Three types of autophagy [macroautophagy, microautophagy, and chaperone-mediated autophagy (CMA)] are depicted in Figure 1.

### 2.1. Mechanisms of Autophagy

Autophagy refers to the process of delivering cytoplasmic or extracellular components to the lysosomes of an animal cell or the vacuoles of plant or yeast cells [113]. The production and maturation of autophagosomes are directly regulated by location, timing, and intensity [114]. The phosphoinositide-binding protein, HS1BP3, is a negative regulator of autophagosome biogenesis that regulates the lipid composition and phosphatidic acid (PA) levels of autophagosome precursor membranes [114]. Increased levels of systemic autophagy have been reported in *Caenorhabditis elegans*, with hormetic heat stress and heat-shock responsive transcription factor (HSF-1) inducing autophagy to improve the survival and proteostasis of the worm [115]. Furthermore, it has been revealed that autophagy is fine-tuned by epigenetic regulation, through histone (coactivator-associated) arginine methyltransferase, CARM1, a novel enzyme that follows histone H3R17 dimethylation (histone H3 methylated at arginine 17) which is an important epigenetic marker of starvation-induced autophagy [116]. In addition, the vitamin D receptor has been shown to modulate autophagy in normal mammary glands and luminal breast cancer cells, suggesting a potential therapeutic link between vitamin D levels and breast cancer risk [117]. There are numerous additional endogenous and exogenous factors that modulate autophagy, such as transcription factors, variation in the amount or concentration of various cytoplasmic biochemicals, damaged organelles, exogenous compounds, and pathogens [6,40,103]; therefore, autophagy mechanisms vary. Autophagy can be divided into macroautophagy, microautophagy, and CMA based on the mechanism by which intracellular materials are delivered into the lysosome for degradation and the molecular structures that target substrates to the lysosomes [3,118,119,120]. Although these pathways are mechanistically distinct, they all carry out degradation via the lysosome [54,118]. Most forms of selective autophagy involve the degradation of specific targets; for example, mitophagy (mitochondria), pexophagy (peroxisomes), aggrephagy (protein aggregates), glycophagy (glycogens), lipophagy (lipids), ribophagy (ribosome), xenophagy (pathogens), and ER-phagy [21,121]. Autophagy is a novel, evolutionarily conserved function of the eukaryotic initiation factor 2 (eIF2α) kinase pathway, which consists of a family of evolutionarily conserved serine/threonine kinases that regulate stress-induced translational arrest and are targeted by virulence gene products [122]. 

#### 2.1.1. Macroautophagy

Macroautophagy is initiated when a portion of cytoplasm containing a cellular organelle is sequestered to form the autophagosome [83]. The autophagosome fuses with the lysosome or late endosomal multivesicular bodies (MVBs) to degrade the materials within it. Atg8 (microtubule-associated protein 1A/1B-light chain 3, LC3, is an Atg8 homolog in humans) was the first autophagosomal protein to be characterized [119]. Macroautophagy can be classified as cargo-specific or non-selective [83,119,123].

Mitophagy has been observed in yeasts when a shift occurs between non-fermentable and fermentable carbon sources, such as glucose, following which the surplus mitochondrial population undergoes mitophagy [120,124]. The first protein identified to cause mitophagy in yeast was Uth1p, a member of the SUN family, which is present in the outer mitochondrial membrane and allows excessive mitochondria to be removed during starvation [125]. The mitochondrial outer membrane protein, Atg32, is a receptor for selective autophagy [126] that is not conserved in mammalian species; instead, FUNDC1 and BNIP3, BNIP3L/NIX, and SQSTM1/p62 act as mitophagy receptors, and are dependent upon hypoxia, erythrocyte maturation, and damage-induced mitophagy, respectively [123,127,128]. Pexophagy is also induced in *Saccharomyces cerevisiae* and *Pichia pastoris* via the Atg36 and PpAtg30 receptors, respectively, when the fungal medium is switched from an oleic acid or methanol to a glucose or nitrogen starvation medium [129,130]. Starvation has also been shown to induce non-selective macroautophagy [9], whereas mitochondrial phospholipids have been demonstrated to be required for autophagy [17]. The machinery required for selective autophagy has been studied extensively using yeast cells, revealing that the cytoplasm-to-vacuole targeting (CVT) pathway is used to specifically transport vacuolar hydrolases into the vacuole of budding yeast cells [131]. A high degree of curvature in the initiating membranes (phagophores or isolation membranes) is a prominent feature of CVT vesicles during mammalian autophagy [132]. 

#### 2.1.2. Microautophagy

After the lysosome has formed vesicles by invaginating and engulfing small sections of the cytoplasm, lysosomal proteases degrade the contents of these vesicles [119]. Microautophagy occurs during the biogenesis of multi-vesicular bodies (MVBs), which deliver soluble proteins to the late endosomes, and relies on electrostatic interactions between endosomal sorting complexes required for transport (ESCRT) I and III and the heat-shock cognate protein 70 (HSC70). Hence, microautophagy involves both endocytic and autophagic components [133,134]. 

#### 2.1.3. Chaperone-Mediated Autophagy (CMA)

**Figure 2:**
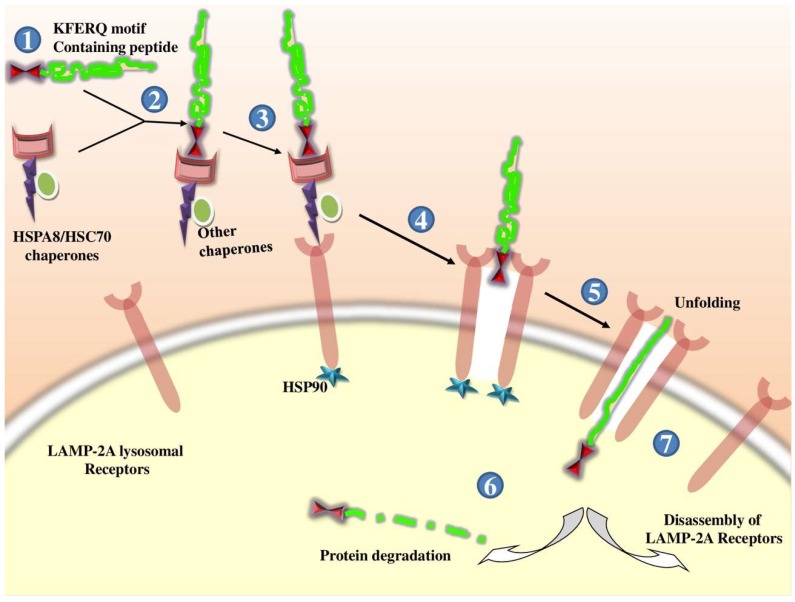
In chaperone-mediated autophagy (CMA), (**1**) KFERQ motif that is present in 30% of soluble cytosolic proteins (**2**) is recognized by cytosolic chaperone protein HSPA8/HSC70, which is present in a complex with other chaperone proteins. (**3**) Such recognized proteins bound to lysosomal receptor protein LAMP-2A. (**4**) Binding of the substrate with the LAMP-2A leads to oligomerization of receptors. (**5**) With the help of HSP90, the substrate is then unfolded and translocated through LAMP-2A-enriched translocation complex. (**6**) After reaching inside the lysosomes, the proteins are degraded (**7**), and the LAMP-2A receptors are disassembled.

Only proteins with a C-terminal pentapeptide KFERQ motif undergo CMA; the HSC70 cochaperone identifies cytosolic proteins containing this sequence and delivers them to the lysosome [135,136]. Chaperones bound to the substrate are transported to the lysosomal surface, where they interact with the monomeric LAMP-2A [137,138]. LAMP-2A must form a multiprotein complex to translocate the substrate [139]; LAMP-2A complex assembly is a dynamic process that occurs when the substrate binds to the receptor. The unfolded substrate protein (chaperon-mediated) is then translocated into the lysosome by LAMP-2A for degradation, following which LAMP-2A disassembles and its monomers are degraded in lipid microdomains. The levels of LAMP-2A tightly regulate the rate of CMA at the lysosomal membrane [15,140]. In the mammalian anti-viral defense system, a cell-autonomous autophagy mechanism has been identified wherein cellular p62 adaptor-mediated autophagic viral protein clearance induces cell survival [141]. Some positive-strand RNA viruses, including picornaviruses and influenza virus, promote autophagic membrane formation, and inhibit their final maturation (lysosomal fusion) [142,143,144]. Consequently, studying the interactions between autophagy and adenoviruses could improve adenoviral-based oncolytic virotherapies [145]. The process of CMA is depicted in Figure 2.

### 2.2. Molecular Mechanisms of Autophagy

Autophagy is an evolutionarily conserved process induced via multiple signaling pathways by numerous stimuli including nutrient starvation [16,105], hypoxia [82,146], oxidative stress [109,147], pathogen infection [39,148], and ER stress [149]. In the presence of nutritional substances and cytokines, mechanistic/mammalian target of rapamycin (mTOR) can prevent apoptosis and stimulate cell growth [150], whereas stress and nutrient starvation inhibit mTOR to initiate autophagy via at least four molecular complexes, including the unc-51-like kinase (ULK) complex, consisting of ULK-1, Atg13, Atg101, and FAK-family interacting protein (FIP200); the PI3K complex, consisting of Atg15, vacuolar protein sorting (VPS)15, VPS34, Beclin 1, and Beclin 1-regulated autophagy protein 1 (AMBRA1) [151,152,153]; transmembrane protein complexes, including Atg9 and WIPI; and two ubiquitin-like protein conjugation systems (Atg12 and LC3) [154,155]. 

Autophagy is initiated by the assembly of the ULK complex, which phosphorylates AMBRA1 and leads to activation of the PI3K complex [155,156]. Class III PI3K is known to participate in various membrane trafficking events, whilst PI3K and Beclin 1 mediate membrane nucleation. The Atg5-Atg12-Atg16 complex is recruited to the pre-autophagosomal structure (PAS) where it associates with the outer membrane of the phagophore, essentially preventing the premature fusion of vesicles and lysosomes [157]. The second ubiquitin-like system stimulates the binding of phosphatidylethanolamine (PE) and Atg8/microtubule-associated protein 1 light chain 3 (LC3). LC3 has a high affinity for the lysosome when bound to the phagosome (LAPosome); thus, any engulfed pathogens will be killed and degraded at a higher rate [158]. Atg4, Atg7, and Atg3 process LC3 into LC3-II, a molecular marker for autophagosomes [86] that is present on both its inner and outer surfaces and is essential for the expansion and completion of the autophagic membrane. Following autophagosomal closure, the Atg5-Atg12-Atg16 complex dissociates from the autophagosome. Atg9 is required for the formation of intraluminal vesicles and is localized within the autolysosome for acidification [159]; Atg9 is also translocated to the site of autophagosome formation where it provides a membrane to elongate the limiting membrane, known as the phagophore [160]. The autophagosome then fuses to the lysosome to form the autolysosome, which is regulated by lysosomal membrane proteins and cytoskeletal proteins [2]. The LAMP-1/2 protein controls autophagosomal maturation. Genetic mutations in LAMP-2 are known to cause Danon disease, a glycogen storage disorder linked to hypertrophic cardiomyopathy, skeletal muscle weakness, and intellectual disability [161]. Within the autolysosome, hydrolytic enzymes digest the internalized cargo and the internal autophagosome membrane, then the digested products such as amino acids are released into the cytosol to be recycled. Autophagosomes are also directly related to cell trafficking pathways. 

Recently, Holland et al. identified that the phosphoinositide-binding protein HS1BP3 negatively regulates autophagosome production [162]. HS1BP3 is thought to reduce phospholipase D1 (PLD1) activity and its localization to ATG16L1 and transferrin receptor (TFRC)-positive vesicles. It is also known to regulate the levels of PA and the lipid content of autophagosome precursor membranes [114]. Two large families of E3 ubiquitin ligases, TRIM and CULLIN, have been recognized as important autophagy regulators which promote or inhibit the process, respectively [81]. The GTPase Ras-related protein in brain 7 (Rab7) also plays a key role in autophagy regulation, particularly in modulating its flux [163]. Knockdown of the small GTPase Rab13 has been shown to inhibit pterostilbene-induced autophagy in vascular endothelial cells (VECs), whilst its upregulation stimulates autophagy in VECs [164]. Under basal autophagy conditions in humans, proteomic analysis of the autophagy interaction network (AIN) revealed a network of 751 interactions between 409 candidate proteins [165]. In order to identify the proteins modulating starvation-induced autophagy, genome-wide screening of siRNA in a GFP-LC3-expressing human cell line was carried out [166], shortlisting nine proteins. One of these, short coiled-coil protein (SCOC), forms an essential starvation-sensitive trimeric complex with UV radiation resistance-associated gene (UVRAG) and WAC (WW domain-containing adapter protein with coiled-coil), which is a negative regulator of the ubiquitin-proteasome system. Genome-wide studies in *C. elegans* identified 139 genes that promote autophagy when inactivated [167]. Long ncRNAs (lncRNAs), which are longer than 200 nucleotides and do not encode proteins, often possess regulatory functions; for example, miR188-3 has been found to regulate Atg7 expression. RNA-linked strategies have revealed several autophagy regulators such as RNA-binding proteins (RBPs), which are post-transcriptional and co-translational regulators with RNA-related functions. Surprisingly, various key autophagy proteins, including LC3B and LAMP-2C, have been found to bind RNA [111]. A considerable amount of autophagy research is being carried out worldwide; however, these innovative findings have raised numerous additional questions. To some extent, autophagy research has been protein-centric, and innovative new approaches have been developed to strengthen this focus in recent years. Among these, genome-wide screens and proteomics-based strategies have revealed substantial interlinking between autophagy and RNAs; however, the precise mechanisms underlying this association require further investigation. Future studies must develop and evaluate novel agents that specifically target the autophagy pathway. 

**Figure 3:**
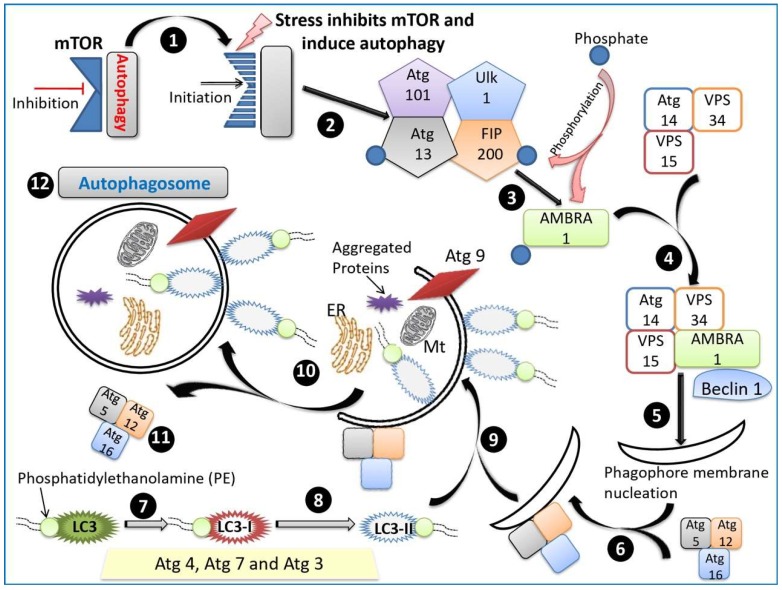
Process of autophagosome formation. (**1**) Autophagy is inhibited by mTOR. (**2**) Various kinds of stress (hypoxia, oxidative stress, pathogen infection, endoplasmic reticulum stress or nutrient starvation conditions) inhibit mTOR, and the process of autophagy is initiated. (**3**) Assembly of ULK complex occurs, and the complex includes ULK-1, autophagy-related protein 13 (Atg13), Atg101 and FAK-**F**amily **I**nteracting **P**rotein (FIP200). (**4**) The complex phosphorylates AMBRA1. (**5**) AMBRA1 activates PI3K complex encompassing Atg15, vacuolar protein sorting 15 (VPS15), VPS34, Beclin-1 and AMBRA1 which helps in nucleation. (**6**) Atg5-Atg12-Atg16 complex is recruited to phagophore and prevent premature fusion of vesicle to the lysosome. (**7**) LC3 is conjugated with PE by the ubiquitin-like system and (**8**) transformed into LC3-II with the help of Atg4, Atg7, and Atg3. (**9**) LC3-II is present on both the inner and outer surfaces of the autophagosome. (**10**) Atg 9 further elongates the membrane and forms intraluminal vesicles; also required for local acidification. (**11**) Atg5-Atg12-Atg16 complex is dissociated from the complete autophagosome (**12**).

A pictorial representation of the process of autophagosome formation is presented in Figure 3.

### 2.3. Autosis: A Novel Form of Autophagy

Liu and Levine [168] described a novel form of non-apoptotic autophagic gene-dependent cell death, termed autosis, which is mediated by the Na^+^/K^+^-ATPase pump. Autosis involves enhanced cell-substrate adhesion, focal ballooning of the perinuclear space, and dilation and fragmentation of the endoplasmic reticulum. The Tat-Beclin 1 peptide complex may initiate autosis, with the fusion of the evolutionarily conserved, 18-amino acid-long Beclin 1 domain with 11 amino acids from the HIV Tat protein transduction domain aiding the cellular entry of the fusion peptide [169]. The Tat-Beclin 1 fusion peptide has been shown to inhibit the replication of HIV, CHIKV, Sindbis (SINV), and WNV, as well as intracellular bacteria such as *Listeria monocytogenes* [23]. Tat-Beclin 1 treatment also reduced mortality in neonatal mice infected with CHIKV and WNV, demonstrated using a TUNEL assay, and cleared mutant Huntingtin protein aggregates [170]. Autosis can be partially rescued by knocking down Atg13 or Atg14 or using 3-methyladenine. Under serum/amino acid starvation, approximately 1% of dying cells exhibit a morphology similar to that of cells treated with Tat-Beclin 1 and autosis is selectively blocked when the Na^+^/K^+^-ATPase pump is inhibited [171]. During cerebral hypoxia or ischemia, the neonatal brain releases cardiac glycosides (ouabain or endobain), which inhibit the Na^+^/K^+^-ATPase pump and reduce autosis [172]. Autosis has also been observed in patients with severe liver anorexia nervosa who display focal ballooning of the perinuclear space, convoluted nucleus, dilated and fragmented ER, empty vacuoles, and several autolysosomes in their hepatocytes [173]. Ischemic injury can also lead to autosis in other organs, including the kidney and heart, which is attenuated in Beclin 1^+/−^ mice [168,174]. 

### 2.4. Association between Autophagy and Other Cell Death Mechanisms

Autophagy can promote or inhibit cell death depending on the cellular context; many other death mechanisms are intricately involved in the processes, with several mechanistic links elucidated between autophagy and other death mechanisms.

#### 2.4.1. Links between Autophagy and Apoptosis

Autophagy and apoptosis regulation overlap when the BH3 domain of the Beclin 1 autophagy protein interacts with anti-apoptotic proteins of the Bcl-2 family, including Bcl-2, Bcl-xL, and Mcl-1 [175,176,177,178]. The BH3 domain has a critical role in the interaction between these proteins and has been shown to interact with the receptor domain of the Bcl-2 family in nutrient-rich cells [178]. Beclin 1-mediated autophagy is inhibited by ER-localized Bcl-2 [179]; the transgenic expression of Bcl-2 was shown to inhibit autophagy in mouse heart muscles. Beclin 1 mutants, which are unable to bind to Bcl-2, induce higher levels of autophagy than their wild-type counterparts [179,180]; hence the physical Beclin 1-Bcl-xL/Bcl-2 interaction regulates Beclin 1-mediated autophagy [179]. ABT737, a compound which mimics the BH3 domain and thus inhibits this interaction, increases the aggregation of LC3, an autophagy marker which is present on autophagosomes [181], in both nutrient-rich and nutrient-deprived media. Furthermore, the knockdown of Beclin 1 and other essential Atg proteins using siRNA heteroduplexes was shown to reduce ABT737-stimulated LC3 aggregation [168]. Atg12 is a dual-functioning protein that participates in both autophagy and apoptosis [108]; non-conjugated Atg12 can bind and inhibit Mcl-1 and Bcl-2 via a BH3-like domain to positively regulate mitochondrial apoptosis. Atg12 knockout inhibits the release of cytochrome c from the mitochondria and apoptosis, whilst abnormal Atg12 expression represses the anti-apoptotic activity of Mcl-1 [182]. 

Autophagy promotes apoptosis by degrading a negative regulator of the Fas ligand [183]; however, it can also protect cells against apoptosis induced by tumor necrosis factor (TNF)-related apoptosis-inducing ligand (TRAIL) by altering the concentrations of Bcl family members [184]. Similarly, components were found to be degraded by autophagy during developmental apoptosis [185], whilst it was recently shown that inhibiting autophagy increased apoptosis and accelerated mortality in murine sepsis models with inadequate autophagy pathways in CD4^+^ T cells, indicating that autophagy has a functional role against apoptosis and immunosuppression in T cells in sepsis [186]. Furthermore, TRAIL combined with a novel chalcone derivative, Chal-24, was found to remarkably increase lung cancer cell cytotoxicity via autophagy-mediated apoptosis [187].

#### 2.4.2. Autophagy and Necroptosis

Necroptosis is often associated with inflammation [63]. The relationship between autophagy and necroptosis is complex, elusive, and slightly controversial since reports have indicated that necroptosis may promote [188], inhibit [189,190], or do not affect autophagy [191]. In several cell lines, including L929 cells, lymphocytes, and cancer cells, autophagy is activated in the presence of TNFα and under starvation to suppress necroptosis [190]. The apoptosis-inhibiting peptide, carbobenzoxy-Val-Ala-Asp (zVAD), prevents autophagy and induces necroptosis in response to TNFα by regulating lysosomal cathepsins, highlighting the pro-survival function of autophagy against necroptosis [192,193]. Similarly, inhibiting the mTOR signaling pathway can prevent apoptosis and even enhance necroptosis, whereas starvation, which induces autophagy, protects cells from zVAD-mediated necroptotic death [194]. 

Sirtuins (SIRT) are NAD^+^-dependent protein deacetylases which are actively involved in both autophagy and necroptosis, as well as transcription, stress resistance, and aging. SIRT-1 deacetylates various components of the autophagy pathway, including Atg5, Atg7, and Atg8 [195], thus promoting autophagy. In cancer cells, dissociation of the FoxO1 transcription factor from SIRT-2 during oxidative stress or starvation results in the acetylation and binding of FoxO1 to Atg7, which subsequently induces autophagy [196]. The binding of SIRT-2 to receptor-interacting protein (RIP) 3 mediates RIP1 deacetylation in response to TNFα; RIP1 and RIP3 then form a complex, which triggers necroptosis [197]. The switch between necroptosis and apoptosis is achieved by recruiting necrosome components to autophagy machinery. Atg5 knockdown reduced the association between RIPK1 and MLKL, suggesting that Atg5 is important in TRAIL-induced necrosome activation. Furthermore, Atg5 knockout in the Atg5^-/-^ DF-1 cell line inhibited autophagy but promoted apoptosis [198]. Autophagy machinery also affects the mechanism of cell death by promoting efficient necrosomal activation and MLKL phosphorylation, thus inducing necroptosis [199]. Several anti-cancer agents, including sorafenib, cause deficient autophagosome formation and facilitate the interaction between p62 and RIPK, resulting in cell death by necroptosis [200]; however, there is still much to be elucidated about the interplay between these two processes. 

#### 2.4.3. Autophagy and Necrosis

Necrosis refers to the increase in cell volume caused by organelle swelling, which results in plasma membrane rupture and the loss of intracellular contents. When ATP is depleted, the cell is unable to undergo apoptosis and undergoes necrosis instead [201]. Poly ADP ribose polymerase (PARP1) is an enzyme with roles in DNA repair, transcriptional regulation, and chromatin modification [202]. PARP1 over-activation decreases the ATP reservoir and induces necrotic cell death by bypassing energy-dependent apoptotic cell death [203]. ATP depletion also activates AMP-activated kinases (AMPK) [204], which induce autophagy by activating the ULK1 complex or inhibiting mTOR signaling [205]. Thus, DNA damage-induced PARP1 activation leads to a decline in ATP levels, AMPK activation, mTOR inhibition, and autophagy induction [206]. PARP1 plays a dual role in autophagy and necrosis since autophagy is a pro-survival mechanism, whilst necrosis is a pro-death mechanism. The fate of the cell depends on the balance between autophagy and necrosis, where autophagy represents the final attempt of the cell to survive before necrosis. 

## 3. Role of Autophagy

### 3.1. Role of Autophagy against Infectious Diseases

#### 3.1.1. Anti-Bacterial Role of Autophagy

**Figure 4:**
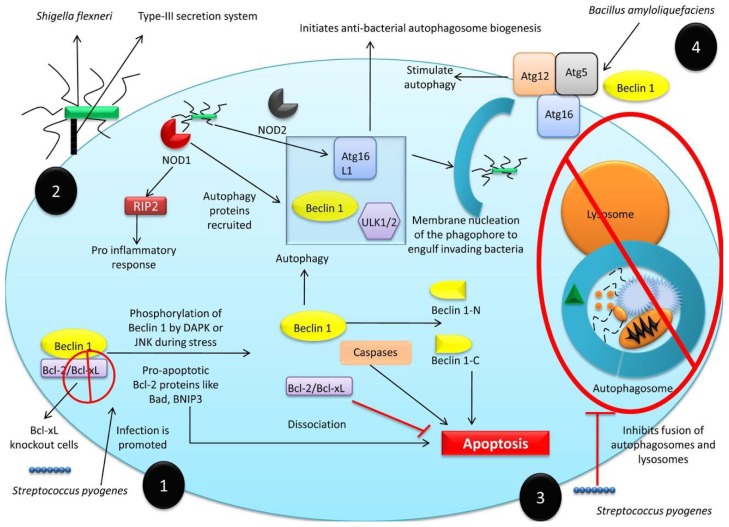
Anti-bacterial role of autophagy. (**1**) Bcl-xL regulates the autophagy, and in Bcl-xL knockout cells, *Streptococcus pyogenes* infection is promoted. (**2**) *Shigella flexneri* invasion in non-phagocytic cells is dependent upon the type-III secretion system (T3SS) effector proteins. Following internalization nucleotide-binding oligomerization domain (NOD)-like receptors (NLRs) detect bacterial peptidoglycans and trigger pro-inflammatory immune response. Bacterial sensing inside the cell either by NLRs or sequestosome-1-like receptors (SLRs) recruits autophagy proteins including unc-51-like kinase (ULK) 1/2 with lipid kinase complexed with Beclin 1 and Atg16L1 to initiate membrane nucleation of the phagophore to engulf invading bacteria. (**3**) Group A *Streptococcus* species inhibits autophagy directly by suppressing the fusion of autophagosomes. (**4**) *Bacillus amyloliquefaciens* was found to stimulate autophagy by elevating the expression of Beclin 1 and Atg5-Atg12-Atg16 complex.

Autophagy plays a beneficial role against infectious diseases by simultaneously degrading pathogens and activating the host immune system [91]. This enables infections to be countered directly, by killing infectious agents, and indirectly, by inducing host immunity against pathogens. Autophagy provides an excellent intracellular defense system against bacterial pathogens, including *Salmonella enterica* serovar Typhimurium [51,207], *Listeria monocytogenes* [50,208], and *Shigella flexneri* [209]. Anti-bacterial autophagy is termed xenophagy [21,53,210]. Numerous cellular, membrane-associated, or cytoplasmic moieties modulate xenophagy; and those cells unable to carry out xenophagy, exhibit higher rates of infection. Bcl-xL regulates xenophagy, and Bcl-xL knockout cells are more susceptible to *Streptococcus pyogenes* infection [53]. The infection of non-phagocytic cells by *Shigella flexneri* is dependent upon type-III secretion system (T3SS) effector proteins [52] which reorganize the host cell cytoskeleton, ruffle the cell membrane, and cause bacterial uptake. Following internalization, bacterial peptidoglycans are detected by nucleotide-binding oligomerization domain (NOD)-like receptors (NLRs) which trigger a pro-inflammatory immune response [211] (Figure 4). The bacteria-sensing NOD proteins interact with Atg16L1 to initiate anti-bacterial autophagosome biogenesis in response to bacterial invasion [212]. Intracellular bacterial sensing either by NLRs or sequestosome-1-like receptors (SLRs) recruits autophagy proteins, including unc-51-like kinase (ULK) 1/2 and lipid kinase complexed with Beclin 1 and Atg16L1, to initiate phagophore membrane nucleation and engulf invading bacteria [213]. 

Mutant *C. elegans* with defective autophagy genes exhibit increased susceptibility to bacterial infection [214]. In addition, it has been reported that HLH-30/TFEB-mediated autophagy and autophagy pathways can regulate the tolerance of *C. elegans* to *Bacillus thuringiensis* infection by protecting against its pore-forming toxins [215], suggesting a novel association between intrinsic epithelial defenses and HLH-30-mediated autophagy against in vivo bacterial attacks. Liang et al. [176] reported that Beclin 1 overexpression inhibits Sindbis virus replication, indicating that autophagy protects against infectious pathogens. Autophagy may activate innate immunity against mycobacteria via pattern recognition receptors (PRRs) or non-receptor-mediated processes [49].

Infection with group A *Streptococcus* species (GAS; *Streptococcus pyogenes*) induces anti-apoptotic Bcl-xL expression which inhibits autophagy directly by suppressing autophagosome-lysosome fusion, and indirectly by interacting with Beclin 1-UVRAG to suppress GAS internalization [53]. In addition, *Mycobacterium tuberculosis* is known to induce miR144 expression in human macrophages and monocytes and adversely affect their antimicrobial activities and innate host immune responses against the bacterial infection by targeting DRAM2 (DNA damage-regulated autophagy modulator 2), which is a critical element of the autophagy response [216]. The ubiquitin ligase, SMURF1, has also been shown to control *M. tuberculosis* replication in human macrophages by associating with bacteria in the lungs of patients with pulmonary tuberculosis. The murine macrophage cell line, RAW264.7, has been used to study *Bacillus amyloliquefaciens* SC06-induced autophagy and its anti-bacterial response against *Escherichia coli*; *B. amyloliquefaciens* stimulated autophagy by increasing the expression of Beclin 1 and the Atg5-Atg12-Atg16 complex, but not activating the AKT/mTOR signaling pathway [217].

Several autophagy-inducing drugs have been used to treat microbial infections; for example, AR-12 [2-amino-N-[4-[5-(2 phenanthrenyl)-3-(trifluoromethyl)-1H-pyrazol-1-yl] phenyl]-acetamide] inhibits phosphoinositide-dependent kinase-1 and eliminates *Salmonella typhimurium* in murine macrophages and *Francisella tularensis* in human leukemic THP-1 macrophages [218,219]. Furthermore, 1α,25-dihydroxycholecalciferol, a form of vitamin D, can enhance autophagy and inhibit human immunodeficiency virus (HIV) replication in macrophages [220].

Many bacteria have evolved mechanisms to overcome autophagy and allow them to replicate within infected cells or even within autophagosomes. These bacteria may express receptors to prevent or enhance phagosome formation, capture nutrient containing phagosomes, subvert autophagy machinery, prevent fusion, or resist autophagy. Certain bacteria hijack autophagosomes and use the by-products of autophagic degradation for microbial replication [221]. *Anaplasma* (formerly *Ehrlichia*) *phagocytophilum*, the causative agent of human anaplasmosis, uses the effector *Anaplasma* translocated substrate 1 (Ats-1) to enhance autophagosome formation and acquire nutrients from inside the autophagosome [222]. After entering the cell, *A. phagocytophilum* replicates inside a double-lipid bilayer membrane associated with LC3 (Atg8), Beclin 1, and Atg6 but lacking lysosomal markers. Inhibiting autophagy with 3-methyladenine did not prevent bacterial internalization but arrested its growth [223], indicating that the autophagic machinery had been subverted to facilitate bacterial proliferation. Another bacterium, *Yersinia pseudotuberculosis*, replicates intracellularly inside specific compartments called *Yersinia*-containing vacuoles (YCVs), which contain autophagy markers; however, YCVs are not acidified and sustain bacterial replication [224]. During *Y. pestis* infection, LC3-I is conjugated with PE to recruit LC3-II, a marker of autophagy progression, to the phagosomal membrane [225]. A similar mechanism is used by *Coxiella burnetii*, the causative organism of Q fever. *Coxiella*-replicative vacuoles contain LC3, Beclin 1, and Rab24; overexpression of these proteins increases the number of *Coxiella*-replicative vacuoles [226]. *Brucella abortus* replicates within *Brucella*-containing vacuoles (BCVs) which traffic from the endocytic compartment to the endoplasmic reticulum, where the bacteria proliferate. Bacterial proliferation requires the autophagy-initiation proteins ULK1, Beclin 1, and Atg14L; however, Atg5, Atg16L1, Atg4B, Atg7, and LC3B are not required [227].

Pathogens such as *Brucella* spp. and *Porphyromonas gingivalis* have evolved to survive inside autophagosomes by preventing its fusion with the lysosome, thus escaping host innate immunity mechanisms [228,229]. *Salmonella typhimurium* studies have revealed that autophagy targets invading intracellular bacterial pathogens for degradation [230]; *S. typhimurium* regulates the SIRT1/LKB1/AMPK complex of the mTOR pathway by targeting SIRT1, LKB1, and AMPK to lysosomes for rapid degradation, restricting autophagy and disrupting AMPK-mediated mTOR regulation [231].

Autophagy is differentially regulated in tuberculoid and lepromatous leprosy [232]; in tuberculoid skin lesion cells, autophagy controls *Mycobacterium leprae*, whereas in lepromatous cells, the blocking of Bcl-2-mediated autophagy promotes bacterial persistence. IFN-γ may counteract the *M*. *leprae*–mediated inhibition of autophagy in lepromatous macrophages as autophagy levels were restored in lepromatous patients who developed the reversal reaction, an inflammatory state associated with augmented IFN-γ and rapamycin treatment, indicating that autophagy is an important innate mechanism associated with *M*. *leprae* control in skin macrophages [232]. 

#### 3.1.2. Anti-Viral Role of Autophagy

Autophagy has a beneficial role in cellular defense against invasion by viruses; therefore, it has been used for antiviral immunity [141,233,234]. Autophagy helps to clear viral pathogens during infection via various molecular mechanisms, regulates immune responses, and prevents harmful overactivation and inflammation [235]. For example, autophagy increases the presentation of endogenous viral antigens in the peptide grooves of major histocompatibility complex (MHC) class I molecules on the cell surface during herpes simplex virus type 1 (HSV-1) infection. Studies of viral peptides have suggested a complex interaction between vacuoles and MHC class I presentation pathways in autophagosomes [236]. In contrast, MHC class II molecules continuously accept input from autophagosomes, which facilitates antigen presentation by MHC class II molecules [237,238]. Autophagy is a major component of *Drosophila* immunity against vesicular stomatitis virus (VSV) [239] as it can deliver viral antigens to TLRs for presentation. During anti-viral signaling, pattern recognition receptors (PRRs) at the plasma membrane (i.e., Toll-7) that are engaged by VSV stimulate an autophagy-dependent innate immune response mediated by PI3K-Akt-signaling [239,240]. Furthermore, Toll/TLR signaling has been shown to regulate the Rift Valley fever virus (RVFV) replication in both flies and mammals [241], whilst SIRT1, an NAD(+)-dependent deacetylase, modulates the activation of dendritic cells and autophagy during induced immune responses against respiratory syncytial virus (RSV) [242], thereby directing an effective anti-viral immune response. Furthermore, autophagy is stimulated by the salicylamide derivatives against cytopathic bovine viral diarrhea virus (cp-BVDV), a Flaviviridae pestivirus [243]. Foot-and-mouth disease virus (FMDV) infection suppresses autophagy and NF-κB anti-viral activities by degrading Atg5-Atg12 using the viral protein, 3C^pro^, suggesting that Atg5-Atg12 positively modulates anti-viral NF-κB and IRF3 pathways during FMDV infection to limit FMDV proliferation [244]. However, autophagy is often hijacked by viral pathogens and can be modulated to their own benefit.

#### 3.1.3. Proviral Role of Autophagy

Subverting the autophagic pathway can have adverse consequences by giving pathogens access to nutrients for growth and reproduction [245]. Autophagy plays an important role in viral replication and pathogenesis [246], with coronaviruses [247], coxsackievirus B3 [248], poliovirus [249], hepatitis C virus (HCV) [142,250,251,252], and DENV [143] all known to stimulate and require autophagy for accelerated replication. For instance, autophagy has been demonstrated to be actively involved in the replication of influenza A virus (IAV), which induces autophagosome formation during the early phase of infection and later inhibits autophagosomal maturation by preventing autophagosomal-lysosomal fusion and promoting autophagosomes to accumulate in virus-infected cells [253]. Autophagy-deficient cells are more susceptible to apoptosis upon influenza infection [253,254], while using pharmacological reagents or RNA interference to alter cellular autophagy can impair viral protein accumulation [255]. Human single-chain antibody variable fragments (ScFvs) which bind to the influenza A virus ion channel protein (M2) and inhibit viral replication [256] were found to restore autophagosome maturation suppressed by the infecting virus (personal communication). It has also been reported that HCV can trigger autophagy via immunity-related GTPase M, which promotes HCV replication [257]. Paramyxoviruses such as Newcastle disease virus (NDV) have been shown to trigger autophagy in U251 glioma cells to enhance viral replication [258]. In addition, modulating NDV-induced autophagy using rapamycin, chloroquine, or small interfering RNAs which target genes critical for autophagosome formation (*Atg5* and *Beclin 1*) affects virus production, suggesting that NDV may utilize autophagy to promote its replication [259]. Human immunodeficiency virus (HIV) uses multiple methods to regulate autophagy and enhance its replication [260,261]. HIV induces the early stages of autophagy but inhibits the later stages which would suppress the production of new virions. The HIV-1 accessory protein, Nef, inhibits autophagosomal maturation by interacting with Beclin 1 [262], whilst the HIV protein Vpr can trigger autophagy in transfected THP-1 macrophages, indicating that autophagy may be involved in maintaining HIV reservoirs in macrophages [263]. HSV-1 [264], Kaposi’s sarcoma-associated herpesvirus (KSHV) [265], and mouse herpesvirus 68 (MHV-68) encode proteins that bind Beclin 1 to prevent autophagy initiation [266]. 

During poliovirus (PV) infection, vesicle acidification, which can mature autophagosomes, has been shown to induce the maturation of virions into infectious particles [267]. One of the most important characteristics of high-risk human papillomavirus (hrHPV) etiopathogenesis is that inhibiting host autophagy could cause cervical cancer via hrHPV [268]. In epithelial cells, flavivirus NS4A-induced autophagy protects infected cells and induces viral replication [269]. Autophagy also plays a critical role in the replication of coronaviruses and the generation of their replicative structures [270]. Coronavirus nonstructural proteins (nsp6) induce the formation of omegasomes and autophagosomes from the ER via an omegasome intermediate [271]. In addition, autophagy has been shown to induce the replication of infectious spleen and kidney necrosis virus (ISKNV) in the Chinese Perch Brain (CPB) cell line, suggesting complex interactions between ISKNV and host cells during viral pathogenesis and for anti-viral treatment strategies [246].

Treating FMDV-infected cells with rapamycin, an autophagy inducer, was shown to increase viral replication, whilst inhibiting the autophagosomal pathway using 3-methyladenine or small-interfering RNAs decreased viral replication [272]. Furthermore, disrupting autophagy using the knockdown approach in hepatitis C virus (HCV)-infected hepatocytes stimulated the interferon signaling pathway and induced apoptosis, indicating that HCV-induced autophagy can impair the innate immune response [251]. Suppressing HCV-induced autophagy could be a promising approach for inhibiting exosome-mediated viral transmission [273], besides autophagy has been shown to reduce HCV clearance following IFN-α/Ribavirin (RBV)-based anti-viral therapy [274]. A DENV study revealed that autophagy inhibitors are better candidate targets than conventional anti-viral therapies using interferons (IFNs) [275]; upregulating cellular autophagy was reported to inhibit RLR-mediated type-I IFN-independent signaling and cause the antibody-dependent enhancement (ADE) of DENV [274]. Suppressing autophagic vacuoles has been demonstrated to stimulate the maturation of infectious bursal disease virus [276]. Adenoviral infection may be favored by autophagy via an increase in ATP, essential to increase anabolism of the infected cells and amino acid pools for the synthesis of viral proteins. In the later stages of adenoviral infection, Atg12-Atg5 complex is significantly upregulated as an evidence of enhanced autophagy [277]; therefore, autophagy may improve the virulence of some viruses. Autophagy genes are involved in the regulation and execution of autophagy [278]. Beclin 1 was the first mammalian gene identified to stimulate autophagy [279]. Some viruses, such as α-, β- and γ-herpesviruses, encode the neurovirulence protein, ICP34.5, which associates with Atg6/Beclin 1 and inhibits autophagy by preventing the formation of the PI3 kinase complex [264]. The autophagy genes *Fip200*, *Beclin 1*, *Atg14*, *Atg16l1*, *Atg7*, *Atg3*, and *Atg5* have been found to promote the reactivation of latent murine gamma-herpesvirus 68 by inhibiting virus-induced systemic inflammation molecules, such as IFN-γ [280]. In contrast, autophagy inhibition has been reported as a new molecular mechanism by which HSV-1 escapes innate immunity, resulting in fatal disease [281]. The autophagic cell death of alveolar epithelial cells has been observed to play a major role in the high mortality rate caused by H5N1 influenza virus infection; hence autophagy-blocking agents could have preventative and therapeutic effects against this virus [282]. Activating the PI3K /Akt/mTOR pathway and inhibiting autophagy have been shown to promote the cellular entry of HPV type 16 [283], contrarily autophagy has been shown to be essential for the replication of coronavirus and mouse hepatitis virus (MHV) [284]. *HSV-1* mutants, those are unable to inhibit autophagy grows to low virus titer and are less pathogenic [264]. Autophagy evokes antiviral adaptive immunity via the endogenous presentation of viral antigens through the MHC class II pathway [278].

**Figure 5:**
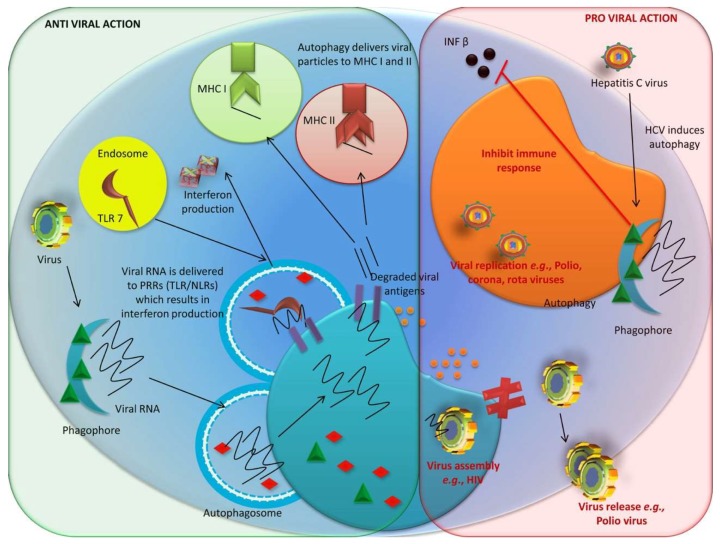
Proviral and anti-viral actions of autophagy.

The proviral and anti-viral actions of autophagy are illustrated in Figure 5.

### 3.2. Autophagy in Tumor Suppression

**Figure 6:**
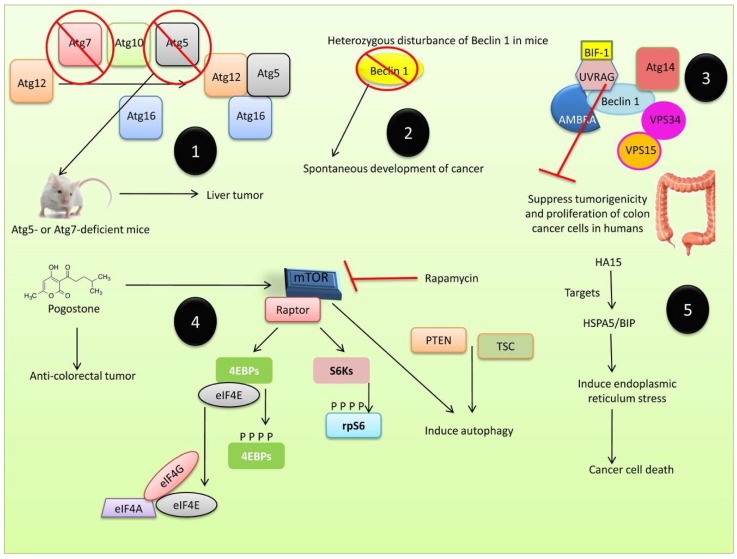
Autophagy in tumor suppression. (**1**) The Atg5- or Atg7-deficient mice showed liver tumors, indicating that defective autophagy can affect the suppression of tumorigenesis. (**2**) Beclin 1 inhibits the growth of tumor in cell lines such as the breast cancer cell line, MCF-7, in which the expression of Beclin 1 was lower than in normal epithelial breast cells. (**3**) UVRAG protein could suppress tumorigenicity and proliferation of colon cancer cells in humans. (**4**) mTOR is implicated in cancer and its substrates include the eukaryotic initiation factor 4E (eIF4E)-binding proteins (4E-BPs) and the ribosomal S6 kinases (S6Ks) 1 and 2, which promote cell cycle progression. The mTOR, which is inhibited by rapamycin, induces autophagy. (**5**) A novel anti-cancer molecule, HA15, which targets HSPA5/BIP was shown to induce endoplasmic reticulum stress and increase the unfolded protein response, resulting in cancer cell death through autophagy and apoptosis.

Initially, autophagy was thought to be involved in tumor suppression by stimulating gene expression, inhibiting proinflammatory mediators, inhibiting inflammation or inflammatory products, and stimulating signaling pathways. The essential *Atg6/Beclin 1* gene was found to be lost monoallelically in 40–75 % of human prostate, breast, and ovarian cancers [285], whereas excessive autophagy stimulation by Beclin 1 overexpression has been reported to inhibit tumor progression [286,287]. Autophagy causes necrosis and chronic inflammation by inhibiting the release of pro-inflammatory HMGB1, which is involved in tumorigenesis [288]. In cell-based assays, inhibiting autophagy was shown to enhance cancer cell growth [289]. p62 (a signaling adaptor/scaffold protein) is involved in the formation of intracellular ubiquitin-related protein aggregates because of autophagy deficiency. Atg7-deficient mice exhibit enhanced accumulation of p62 and ubiquitinated protein aggregates in hepatocytes and neuron [290]. Autophagy has also been implicated in benign hepatomas [291], and the inactivation of Beclin 1 and Atg5 was shown to increase the incidence of cancer in mice [292]. Atg5- and Atg7-deficient mice exhibited liver tumors, indicating that defective autophagy can affect the suppression of tumorigenesis [292]. Heterozygous Beclin 1 (beclin 1+/- mutant) was shown to have a high incidence of spontaneous tumors [289,293], whilst Beclin 1 inhibited tumor growth in cell lines such as the breast cancer cell line, MCF-7, in which Beclin 1 expression was lower than in normal epithelial breast cells [294]. The UVRAG protein was found to suppress the tumorigenicity and proliferation of human colonic cancer cells [295] (Figure 6).

Autophagy suppresses tumor formation by preventing inflammation, the accumulation of proteins and organelles damaged by necrosis, and cellular transformation caused by gene instability [296,297,298,299]. The conserved protein kinase, mTOR, has been implicated in cancer since its substrates (eukaryotic initiation factor 4E (eIF4E)-binding proteins (4E-BPs) and ribosomal S6 kinases (S6Ks) 1 and 2) promote cell cycle progression [299]. mTOR, which is inhibited by rapamycin [300], and molecules such as phosphatase and tensin homolog (PTEN) and tuberous sclerosis (TSC) (products of tumor suppressor genes) can induce autophagy [301,302]. Pogostone, a medicinal herb widely used to treat gastrointestinal diseases, was shown to possess anti-colorectal tumor activities by stimulating autophagy and apoptosis via the PI3K/Akt/mTOR axis [303]. In addition, the novel anti-cancer molecule HA15, which targets HSPA5/BIP, was shown to induce ER stress and increase the unfolded protein response, resulting in cancer cell death via autophagy and apoptosis [304]. Trichosanthin (TCS), a 27 kDa protein from the bioactive component of the root tuber of *Trichosanthes kirilowii* (Chinese cucumber plant; Gua Lou in Mandarin), also exhibited anti-cancer properties against different human ovarian cancer cells via a pathway common to both autophagy and apoptosis [305].

### 3.3. Autophagy in Tumor Progression

Various factors and mechanisms are involved in autophagy-mediated tumor progression. Tumor-induced nutrient shortage, cell debris (degraded proteins), inflammation, and oxidative cascades are all molecular mechanisms affecting tumor progression. During nutrient starvation, autophagy induction promotes the survival of normal cells and may also promote tumor cell survival; however, hypoxia, metabolic stress, energy shortage, oxidative stress-damaged mitochondria, and organelles can be caused by cancer-causing genes or cancer treatments [306]. The undifferentiated colon cancer cell line, HT-29, and other transformed cells have shown an increased tendency to degrade autophagic proteins [287,307,308]. The elevated expression of the autophagy signature protein, BNIP3, a pro-apoptotic Bcl-2 member, has been demonstrated in colorectal and gastric epithelial carcinomas, suggesting that BNIP3 expression may be important for the development of these cancers [309]. The activation of autophagy and peroxisome proliferator-activated receptor gamma (PPARγ) was shown to protect colon cancer cells against apoptosis induced by the interaction between butyrate and docosahexaenoic acid (DHA) in a cell type-dependent manner [310]. Additionally, an Atg8/LC3 family member implicated in autophagy and tumor suppression was associated with alterations to cell death and cytokine secretion in mice lacking gamma-aminobutyric acid receptor-associated protein (GABARAP) [311]. It has been well documented that inhibiting autophagy in cancer cells increases their death, with this strategy proving most useful in tumors that behave like RAS-activated tumors [312]. Inhibiting autophagy is expected to cause ubiquitinated proteins to accumulate and p62 levels to increase; in hepatic tumors, autophagy suppresses spontaneous tumorigenesis via cell-intrinsic pathways whilst p62 accumulation promotes tumor formation [291]. 

The elimination of damaged organelles via autophagy may allow cancer cells to survive despite the stress caused by chemotherapeutic agents [313]. It has also been reported that upregulating autophagy after chemotherapy causes cancer cells to enter a dormant state, which may then propagate at a later stage [314,315]. The state of cell cycle arrest, termed senescence, has been postulated to underlie autophagy-induced tumor cell dormancy [316]. Ras-induced senescence is mediated by autophagy, with autophagy inhibition delaying senescence [317]. Moreover, it has been reported that PSMD10/gankyrin stimulates autophagy in hepatocellular carcinoma (HCC) in response to starvation or stress. A physical association occurs between PSMD10 and Atg7, and is translocated to the nucleus to bind to ATG7 promote and upregulates Atg7 expression [318]. 

### 3.4. Autophagy in Brain Development

There is growing evidence that autophagy performs both physiological and pathological functions in the nervous system, and that autophagic stimulation plays critical roles in neuronal survival and activity. Autophagy is the only method by which neurons degrade and excrete expired organelles, and is responsible for clearing abnormal intracytoplasmic contents from normal cells which would otherwise cause protein accumulation and damage neuronal activity, inducing severe functional impairment. Autophagy also clears protein aggregates from old neurons; thus, inhibiting autophagy can lead to neuronal degeneration and intraneuronal protein accumulation. Mutations in Atg5 confined to neural tissues can cause impaired growth, progressive motor and behavioral deficits, prominent neurodegeneration, and axonal swelling in regions of the brain with increased levels of ubiquitinylated proteins, indicating that autophagy has a neuroprotective role [319]. In mammals, the absence of Atg59 and Atg710 can cause severe neurodegeneration, further supporting the neuroprotective role of autophagy [100,320]. Furthermore, neuronal death can be attributed to the loss of basal autophagy or an imbalance in autophagic flux. In some neurodegenerative diseases, such as Alzheimer’s, Parkinson’s, and Huntington’s, as well as in the brain or spinal cord trauma, the damaged neurons exhibit abnormally high numbers of autophagosomes. Therefore, understanding the interaction between pathophysiological mechanisms and autophagy could be a promising approach for therapies against neurological disorders [321]. Prenatal alcohol exposure has been shown to increase the number of autophagic vacuoles in the cortical micro-vessels of human fetal and mouse neonatal brains, impairing autophagy [322]. Furthermore, autophagy can modulate Notch degradation, stem cell development, and neurogenesis [323].

### 3.5. Autophagy in Neurodegeneration

Several reviews have evaluated the relationship between autophagy and neurodegenerative diseases [324,325]. Autophagy is vital for neuronal homeostasis [326], and its deregulation is highly associated with numerous neurodegenerative effects, such as the accumulation of damaged and toxic molecules with pathological consequences in neurodegenerative disorders such as Alzheimer’s, Parkinson’s, and Huntington’s diseases [327,328]. Lysosomal system inactivation is responsible for the accumulation of autophagosomes observed in Alzheimer’s disease [329], whilst the disease is thought to be due to either excessive or impaired autophagosomal degradation, or the activation of autophagy genes in response to temporary injury/stress in neuronal tissues. Alzheimer’s [330], Parkinson’s [331,332], and Huntington’s diseases [333] are key examples where autophagosomal accumulation and anomalies in the endosomal-lysosomal pathway have been observed in post-mortem human brain tissues via electron microscopy. Autophagy deficiency resulted in neuronal loss in the cerebral and cerebellar cortices in a mouse model [334]. Dysfunction or abnormalities in autophagy, including mutations in autophagy-regulating genes, are accompanied by neurodegenerative diseases across the age spectrum with exceptional frequency. Atg7-deficient mice exhibited ubiquitin accumulation in their CNS, causing nervous symptoms, neurodegeneration, and ultimately death [31,334], whilst Atg5-deficient mice developed cytoplasmic inclusions and exhibited motor dysfunctions [319], and AMBRA 1-deficient mouse embryos displayed neuronal tube defects [335]. 

Autophagy is involved in the cytoplasmic clearance of α-synuclein (α-syn), which is observed in Parkinson’s disease [336]. In a mouse model, Beclin 1 overexpression was found to reduce the clearance of α-syn, leading to pathological neuron abnormalities [337]. Pharmacological and genetic pathways are involved in the degradation of α-syn by polo-like kinase 2 via autophagy, suggesting that these two proteins are concomitantly co-degraded [338]. 

The *PINK1* and *Parkin* genes regulate mitophagy [339], indicating that mutations in these genes can cause defects in mitophagy which have been correlated with Parkinson’s disease [340,341]. Beclin 1 expression is lower in the brains of patients with Alzheimer’s disease and not only affects autophagy but also increases the deposition of β-amyloid proteins causing neurodegeneration [330]. Huntington’s disease is caused by the extension of the polyglutamine (polyQ) proteins aggregate intracellularly, which causes neuronal death. *Atg*-knockout *C. elegans* exhibit increased polyQ toxicity [342], whilst in *Drosophila* the autophagy-enhancing small molecule 2-(4-phenylphenyl)-5,6-dihydroimidazo[2,1-B][1,3] thiazole, also known as autophagy enhancer-99 (AUTEN-99), has been shown to prevent the symptoms of neurodegenerative diseases [343]. The dual role observed for autophagy may be due to our poor understanding of this ubiquitous cellular recycling system. The differences between physiological and pathological autophagy may help design therapeutic strategies specifically targeting pathological autophagy without hindering its physiological roles [344]. For example, the apoptosis-stimulating protein p53-2 (ASPP2/53BP2L) was reported to have different effects on autophagy in neurons stimulated with different levels of gp120, a soluble envelope glycoprotein of HIV-1 that interacts with chemokine receptors such as CXCR4 and CCR5. Thus, regulating autophagy in the CNS could be a potential therapeutic approach against HIV-associated neurocognitive disorders [77].

### 3.6. Autophagy in the Immune System and Autoimmune Diseases

#### 3.6.1. Autophagy in the Immune System

The roles of cellular autophagy in immunological processes and autoimmune diseases have been reviewed extensively [82,345,346]. Autophagy plays important roles in both innate and adaptive immunity [347], modulates cellular and humoral immune responses [348,349,350], and has roles in the non-metabolic and metabolic functions of immune cells [349]. Furthermore, autophagy is involved in innate immune cell differentiation, degranulation, phagocytosis and extracellular trap formation involving neutrophils, eosinophils, mast cells, and natural killer cells, and plays an essential role in the renewal, differentiation, and homeostasis of immune cells [351]. Autophagy also regulates the functional responses of immune cells, such as phagocytosis, antigen presentation, cytokine production, control of inflammasome activation, tolerance, and their consequences on overall host defense via monocytes, macrophages, dendritic cells, and antigen presentation [350]. Additionally, autophagy plays important roles in B cell development, activation, and differentiation, which enables B cells to adapt to various events, and determines their fate, survival, and function [352]. Since B cells produce antibodies, autophagy can determine humoral immune responses. In one study, the B cells of Atg5-deficient mice had defective antibody responses, indicating that autophagy has a role in antibody production [353].

##### Pro-Inflammatory Signaling Regulated by Autophagy

Several studies have documented interplay between autophagy and the NF-κB signaling pathway. Members of the NF-κB family of transcription factors regulate the transcription of genes involved in cell proliferation, survival, differentiation, and development, whilst activation of the inhibitor of NF-κB (IκBα) kinase complex is essential for autophagy induction. T-cell receptor-mediated NF-κB activation in B-cell lymphoma/leukemia is linked with the autophagy adaptor p62/SQSTM1 [354], which modulates the NLRP3-inflammasome activation and IL-1β production in macrophages [355].

##### Interplay between Cytokine Secretion and Autophagy

IL-1α secretion is enhanced in Atg5-deficient macrophages, whilst inhibiting autophagy results in IL-1β overexpression [356]. The anti-inflammatory cytokine, IL-10, inhibits autophagy by activating mTOR complex 1 [357] and inhibits starvation- and IFN-γ-induced autophagy via Bcl-2 and Beclin 1 in various autoimmune and inflammatory disorders [358].

#### 3.6.2. Autophagy and Autoimmunity

Autophagy has predisposing, pathogenic, and therapeutic roles in autoimmunity. Defects in autophagy pathways and/or autophagy-related genes have been implicated in numerous autoimmune and autoinflammatory diseases, including multiple sclerosis, systemic lupus erythematosus (SLE), rheumatoid arthritis, psoriasis, psoriatic arthritis, inflammatory bowel disease, diabetes mellitus, Crohn’s disease, and vitiligo [82,358,359,360]. Abnormalities in the maintenance of homeostasis via autophagy result in the accumulation of dysfunctional or defective cellular organelles, abnormal proteins, infectious agents, and metabolite accumulation. This predisposes cells to the generation of autoantibodies and proinflammatory mediators and exposes vital and susceptible cellular structures to deleterious agents that can cause disease [358,359,360,361]. A recent study revealed a correlation between the expression pattern of autophagy-related genes and the type of lupus nephritis (LN); thus, autophagy could indicate of the type of LN when formulating a treatment regimen [362]. 

Several Atgs are known to be involved in autoimmune disorders, including Atg5, PR domain zinc finger protein 1 (PRDM1; also known as BLIMP-1), and DNA-damage regulated autophagy modulator 1 (DRAM1) in SLE patients and Atg16L1 and immunity-related GTPase M (IRGM) in Crohn’s disease and ulcerative colitis. Autophagy defects have been observed in T cells, B cells, and macrophages [363]. MHC class II antigen presentation by macrophages occurs via CMA; lysosomal proteins have a central role in antigen processing, which is essential for a correct immune system function. Studies in MRL/lpr mice which develop a full panel of lupus autoantibodies revealed that increased lysosomal pH might be an important lysosomal malfunction involved in autoimmunity, and that perturbed lysosomal turnover may lead to hyperactive antigen presentation by antigen presenting cells (APC) in autoimmune disorders [363]. 

In innate immunity, reduced Atg5 and mTOR expression result in defective autophagy, affecting the clearance of dead cells, increasing levels of nucleic acid remnants and self-antigens, increasing type 1 IFN by DCs, and inducing B cell hyper-differentiation and autoantibody production [82,346,358]. It has also been reported that autophagy-related gene knockdown can have therapeutic effects on autoimmune diseases [357]. Modulating autophagy can manage immunity-related and inflammatory diseases [82,345,347,364] by regulating cytokine and antibody production against immunogenic insults to prevent autoimmune diseases. Therefore, regulating autophagy has clinical potential in cancer immunotherapy [365], whilst autophagy and adenoviral combinations are proving beneficial in adenoviral-based oncolytic virotherapy [145].

### 3.7. Autophagy in Cardiovascular Diseases

Under normal conditions, the myocardium exhibits low levels of autophagy, whilst stressful conditions can increase the level of autophagy to increase cell survival [366,367]. Patients with congestive heart failure, coronary artery disease, hypertension, and aortic valvular disease display increased autophagosomal accumulation in their myocardial biopsies [368]. Autophagy levels vary in normal and affected or stressed hearts, with constitutive autophagy maintaining normal structure and function, and upregulated autophagy occurring during cardiac disease or stress [369]. In *Atg5*-deficient mice, contractile dysfunction and hypertrophy have been observed during cardiomyopathy [370], whilst cell culture studies have revealed that autophagic gene deficiency can cause the accumulation of unwanted proteins and contribute to myocardial disease [371]. Similarly, *LAMP-2*-deficient mice displayed increased autophagic vacuole accumulation and could not degrade proteins, thereby promoting cardiomyopathy [371,372]. It has been postulated that during early life, between birth and suckling, autophagy provides the energy required for cardiac cells [373], whilst mitophagy protects cardiac muscles under ischemic stress [374]. 

Increased autophagy can cause heart failure [375], with autophagy-induced degeneration resulting in the death of cardiomyocytes. This knowledge has helped our understanding of the pathogenic role of autophagy in cardiac failure models and helped devise therapeutic targets [376,377]. Autophagy can cause myocardial cell damage via PARP1, which promotes autophagy in cardiomyocytes by modulating FoxO3a transcription [378]. Increased autophagy causes pathological remodeling of the heart, whilst decreased autophagy reduces remodeling [88]. Thus, it has both protective and destructive roles in the cardiovascular system.

### 3.8. Autophagy in Iron Homeostasis

Iron homeostasis involves a form of macroautophagy known as ferritinophagy, wherein ferritin, an iron storage protein, is degraded in the lysosome [379]. Iron levels are tightly regulated in cells; nutrient deficiency induces autophagy, during which cellular proteins and organelles are engulfed by the autophagosome, which then fuses with the lysosome. The degradation of these contents provides essential resources that either promote cell survival or lead to cell death. Iron (Fe), copper (Cu), zinc (Zn), and aluminum (Al) react with molecular oxygen to produce reactive oxygen species (ROS) and reactive nitrogen species (RNS). Besides acting as a cofactor for metalloprotein enzymes involved in redox reactions, iron also plays a major role in mitochondrial ATP metabolism and other cellular processes. The Fenton and Haber-Weiss redox reaction is highly involved in ROS production and Alzheimer’s progression [380]. To maintain iron homeostasis, storage and recycling are critical. When engulfed by macrophages, the iron within erythrocytes is either stored as a ferritin complex or exported from the cell by the ferroportin iron-exporter [381]. 

HSP70 and ferritin bind iron in the cytosol and autophagocytosis of these proteins can sequester redox-active iron in the lysosomes [382]. Cells rich in these proteins exhibit increased resistance to oxidative stress; therefore, autophagy plays a major role in maintaining cellular redox status [383,384]. The autophagy inhibitor NCOA4, which is a substrate of PI3K, has been shown to physically bind to the ferritin protein complex and direct it to autolysosomes for degradation [385]. NCOA4 knockdown prevents the localization of ferritin in the lysosomes and increases the levels of iron-responsive element–binding protein 2 (IRP2), which is a free intracellular Fe antagonist that prevents cell death via exogenous ROS [386]. NCOA4 also acts as an autophagy receptor for ferritin and delivers it to the lysosome to maintain iron homeostasis. Experimentally simulating low-iron conditions by chelating iron revealed that ferritin is degraded to release the stored iron [387].

### 3.9. Autophagy in Obesity and Diabetes

Autophagy is involved in obesity [388] and diabetes mellitus [389]. Improper lipid and glycogen processing can affect the liver activity and thus, insulin synthesis, resulting in diabetes. Studies have shown that hepatocytes from mouse models of obesity display reduced autophagy [390], with decreased Atg7 expression causing ER stress and affecting insulin signaling [390]. Mutant *Atg7* mice also exhibit reduced β cell mass, reduced insulin circulation, and glucose intolerance, indicating that autophagy defects can reduce insulin levels and cause hyperglycemia [391,392]. A genetic mosaic screen for mutations that increase lysosomal and/or autophagic activity in *D. melanogaster* larva revealed that autophagy-lysosome pathways underlie novel cytoprotective features in *Drosophila* [393]. Obesity impairs autophagy in the liver via *S*-nitrosylation, a process induced by nitric oxide (NO). *S*-nitrosylation of the lysosomal enzymes cathepsin B (CTSB) and hexosaminidase subunit β (HexB) impairs normal lysosomal functioning and is carried out by denitrosylation enzymes, particularly *S*-nitrosoglutathione reductase (GSNOR) and thioredoxin [394]. Obesity inhibits the denitrosylation ability of the liver, impairing hepatic autophagy and insulin resistance [395]. In obese animals, hepatic insulin signaling is impaired by NO-induced hepatic autophagy repression, which ultimately causes the progression of type 2 diabetes [396].

The potential roles of autophagy in ameliorating diseases while maintaining homeostasis have been enumerated in Table 1. Details of applied/granted patents for treating autophagy-related ailments and dysfunctions are presented in Table 2.

### 3.10. Diseases Caused by Autophagy Gene Defects

Maintaining homeostasis is the most important role of autophagy, which is the final survival mechanism used to escape cell death. Mutations or genetic dysfunctions in autophagy-associated genes can have a variety of pathological consequences, as described below:

#### 3.10.1. Static Encephalopathy of Childhood with Neurodegeneration in Adulthood (SENDA)

SENDA is a recently discovered type of neurodegeneration associated with iron accumulation in the brain [422]. It begins with early-onset spastic paraplegia and mental retardation during childhood, followed by symptoms of Parkinsonism and dystonia during adulthood along with eye movement abnormalities, dysautonomia, and sleep disorders. Whole-exome next generation sequencing has revealed that SENDA is associated with a mutation in the *WIPI4* gene (also known as *WDR45*), located at Xp11.23 [399,423,424]. The disease is characterized by iron accumulation in the globus pallidus [425]. WIPI4, which is the mammalian homolog of yeast Atg18, is involved in autophagosome formation [426] by binding phosphatidylinositol 3-phosphate, which is recruited to the autophagosome formation site [427]. A severe decline in WIPI4 expression has been reported in lymphoblastoid cell lines derived from patients with SENDA [424]. As the *WIPI4* gene is found on the X chromosome, one of which undergoes inactivation in females, the loss of WIPI4 function is expressed in a mosaic pattern in females. 

#### 3.10.2. Crohn’s Disease

Crohn’s disease is a major inflammatory bowel disease whose symptoms include abdominal pain, diarrhea, vomiting, and weight loss. *Atg16L1* mutations have been found to be linked with Crohn’s disease [428]. Atg16L1 forms a complex with Atg12-Atg5 which initiates autophagosome formation by inducing the conjugation of LC3 with PE [429]. A study on the 300T > A *Atg16L1* mutation revealed that the mutation is differentially involved in Crohn’s disease and canonical autophagy [430]. In mice, the mutations that eliminated or reduced *Atg16L1* expression indicated a relationship between *Atg16L1* mutations and Crohn’s disease [429]. Other autophagy-associated proteins, including IRGM-101, 102, and 103 and NOD2-104, 105, and 106 have also been linked to Crohn’s disease in humans [431]. Autophagy is thought to be involved in the pathogenesis of Crohn’s disease, but it can also control disease severity by reducing the levels of inflammatory mediators [401].

#### 3.10.3. Hereditary Spastic Paraparesis (HSP)

HSP is a neurodegenerative disorder that causes axonal degeneration in the corticospinal or pyramidal motor and sensory tracts that control distal organs and is caused by a recessive mutation in the *TECPR2* gene. TECPR2 interacts with six human Atg8 orthologs to positively regulate autophagy, with *TECPR2* knockdown in HeLa cells reducing autophagic activity and indicating its role in autophagy [432]. The zinc-finger protein spastizin interacts with the Beclin 1-UVRAG-Rubicon complex and is involved in autophagosome maturation. In fibroblasts derived from patients with HSP, knockout of the spastizin gene reduced autophagy and caused autophagosome accumulation by impairing lysosomal fusion [433]. The *ZFYVE26/SPASTIZIN* and *SPG11/SPATACSIN* mutations were recently associated with HSP [434]; however, there are currently no treatments available to reverse nerve degeneration in HSP, with efforts instead directed towards reducing symptoms by physiotherapy and improving spasticity using medication.

#### 3.10.4. Danon Disease

Danon disease is a rare cardiomyopathic disease caused by LAMP-2 deficiency and characterized by the accumulation of glycogen and autophagic vacuoles in cardiac and skeletal muscles, cardiomyopathy, and intellectual dysfunction [435]. The *LAMP-2* gene is alternatively spliced into three isoforms (LAMP-2A, -2B, and -2C) which have different functions in autophagy. LAMP-2A acts as a receptor for CMA, in which proteins carrying the KFERQ motif are selectively trafficked into the lysosome and degraded. LAMP-2B has been suggested to play a prominent role in macroautophagy and is responsible for the Danon phenotype. LAMP-2B-deficient mice exhibit higher mortality and autophagic vacuole accumulation in the liver, kidney, pancreas, and cardiac and skeletal muscles [372]. Whole genome sequencing identified a nonsense mutation (codon 520C>T in exon 4) in *LAMP2* in a family with Danon disease [436]. LAMP-2 knockout resulted in failed autophagic progression, as evidenced by inappropriate cathepsin D processing in the autophagic vacuoles and abnormally high numbers of mannose-6-phosphate receptors that limited the degradation of long-lived proteins during starvation [437].

#### 3.10.5. X-Linked Myopathy with Excessive Autophagy (XMEA)

XMEA is a rare X-linked recessive skeletal myopathy caused by a single-nucleotide substitution (c. 164-7T > G) in *VMA21*, whose protein product assembles proton pumps in the lysosome which generate and maintain the acidity required for the activity of various lysosomal hydrolases [438]. Impaired hydrolase activity can block the final step of autophagy or induce autophagy by inhibiting mTOR. If autophagy is impaired, a feed-forward pathogenic loop is activated, which results in the accumulation of autophagolysosomes containing incompletely-digested products [439]. XMEA is characterized by progressive muscle weakness, particularly in the proximal muscles of the legs, and muscle degeneration (atrophy) in adulthood. Recently, two *VMA21* non-coding microdeletions [440], one intronic (c.54-16_54-8del) and the other in the 3′UTR (c.*13_*104del), were reported to result in more severe clinical manifestations with extra-ocular and upper extremity involvement and earlier disease onset. Besides long-lived proteins and membranes, XMEA also affects membrane repair, interrupts sarcolemmal membrane homeostasis, and increases serum creatine phosphokinase (CPK) levels [440].

#### 3.10.6. Sporadic Inclusion Body Myositis (sIBM)

sIBM is an age-related progressive muscle disorder which presents in the elderly and is characterized by the presence of autophagic vacuoles and ubiquitinylated misfolded multiprotein aggregates. Two major lysosomal proteases, cathepsins D and B, exhibit decreased activation in sIBM muscle fibers [441] whilst LC3-II is increased, indicating that autophagosome formation is increased due to reduced cathepsin D and B activity during autophagosome maturation [442]. T cells may have a role in sIBM [443], whilst allelic variants of the HLA-DR3 locus, other genetic factors, and the HLA genotype are also thought to play major roles in the progression and severity of the disease [444]. Dysphagia (difficulty in swallowing) may occur due to weakness in the neck muscles of patients with sIBM, whilst myalgia (muscle pain) and difficulty in manipulating objects may occur due to weakness in the fingers. 

An overview of diseases caused by genetic defects in autophagy genes is presented in Table 3.

## 4. Treatment of Autophagy-Associated Diseases

To alleviate autophagy-associated diseases, a variety of drugs, biomolecules, chemicals, and epigenetic strategies have been developed to either promote or inhibit autophagy. 

### 4.1. Strategies to Inhibit Autophagy

Autophagy can be suppressed during any stage of autophagic flux. The pro-apoptotic role of autophagy may lead to hyperstimulation-induced excessive activation, which results in detrimental self-cannibalism that can go beyond the limit of cellular recovery. Numerous chemical inhibitors have been identified and tested in various cell and animal models. Most autophagy inhibitors are poorly selective, limiting their wider applications.

#### 4.1.1. Vacuolar-Type H (+)-ATPase Inhibitors

3-methyladenine (3-MA), a class-III PI3K inhibitor, is commonly used to inhibit autophagy [460]. Class III PI3K and Beclin 1 are essential during the first step of autophagy induction [461]; thus, inhibiting PI3K reduces autophagosome formation. Bafilomycin A1 is a vacuolar H^+^-ATPase inhibitor, which functions at concentrations as low as 1 nM to inhibit both the early and late stages of autophagy by activating the mTOR pathway [462]. In early 1998, bafilomycin A1 was reported to inhibit autophagic vacuoles in rat hepatoma [463] by dissociating the Beclin 1-Vps34 complex and preventing autolysosome formation, thus attenuating functional autophagy. Bafilomycin A1 has been tested in a mouse model and found to be safe [464]. Furthermore, disruption of the vacuolar-type H^+^-ATPase complex in mouse liver cells has been shown to induce the rapamycin complex 1 (mTORC1)-independent aggravation of autophagic vacuoles and lysosomes [465]. Concanamycin A belongs to the same class of vacuolar H^+^-ATPase inhibitors as bafilomycin A1 and in the presence of autophagosomes causes autophagic bodies to accumulate in the central vacuole and cytoplasm of tobacco BY-2 cells [466]. Therefore, vacuolar-type H^+^-ATPases have been proposed as potential therapeutic agents. 

#### 4.1.2. Cycloheximide

Cycloheximide is a protein biosynthesis inhibitor that is frequently used in biomedical research. In mouse pancreatic acinar cells, cadmium chloride- or hyperosmotic sucrose-stimulated autophagy was found to be inhibited by cycloheximide [467], which likely inhibits autophagy at the segregation step. Cycloheximide is used to prevent the autophagy-lysosome pathway [468] and its effects can be rapidly reversed by its removal [469].

#### 4.1.3. Lysosome Alkalizers

Chloroquine, hydroxychloroquine, NH_4_Cl, and neutral red are chemicals that can rapidly neutralize the acidic environment of the lysosome; therefore, they are used to block autophagosome maturation. Chloroquine and hydroxychloroquine are repurposed drugs that have been used to treat malaria, SLE, and rheumatoid arthritis [470]; however, higher hydroxychloroquine concentrations are required to induce active autophagy as a cancer treatment, which are usually not achievable in cancer patients. Chloroquine-mediated lysosomal dysfunction is thought to have increased anti-cancer functions when combined with nutrient deprivation [471]. Lys01, a dimeric form of chloroquine in which each molecule is separated by the spacer molecule N-bis(2-aminoethyl)-methylamine, has been reported to inhibit autophagy at a level 10-fold higher than chloroquine Lys05, a water-soluble salt of Lys01, effectively accumulates within the lysosome to concurrently deacidify and inhibit autophagy [472].

#### 4.1.4. Acidic Protease Inhibitors

Lysosomal proteases are active at an acidic pH. The protease inhibitor leupeptin inhibits cysteine, serine, and threonine peptidases to block protein degradation and thus inhibits the final step of autophagy, as evidenced by the accumulation of vacuolar autolysosomes [473]. Cathepsins are aspartic, cysteine, and serine proteases that are transported to lysosomes via mannose-6-phosphate receptors [474] and can be inhibited by the lysosomal protease inhibitors E64d and pepstatin A. Cathepsins B, H, and L are inhibited by E64d, whilst cathepsins D and E are inhibited by pepstatin A. Cells treated with E64d and pepstatin A exhibit increased LC3-II levels [475]. 

Bafilomycin and chloroquine, which inhibit autophagy by targeting lysosomes, have been reported to increase levels of the autophagosome marker LC3-II, block key aspects of autophagy, decrease mitochondrial quality, and increase mitochondrial DNA damage in primary neurons [476].

#### 4.1.5. Genetic Modifications

The *Atg* genes are crucial in autophagy; therefore, the inactivation or knockdown of these genes may be a useful way to manipulate the process [477]. MiR-30a has been reported to downregulate Beclin 1 and Atg5 expression, whilst miR-101 can inhibit basal and rapamycin-induced autophagy. Autophagy is induced by the activation of the ULK complex, which consists of ULK1/2, Atg13, FIP200, and Atg101. In melanoma cells, miR-290–295 clusters can inhibit ULK1 and Atg7 expression to suppress autophagic cell death [478]. MiR-885-3p targets ULK2 to regulate autophagy [479], whereas miRNA-30a/b, miRNA-376b, miR-216a, miR-519a, and miR-17-5p inhibit Beclin 1 expression to suppress vesicle nucleation [480,481,482]. Recently, the leucine-rich repeat kinase 2 gene (*LRRK2*), which is associated with Crohn’s disease, Parkinson’s disease, and leprosy, was shown to inhibit the non-canonical autophagy cascade, indicating that the negative regulation of this cascade could directly induce disease [483]. In addition, the Atg16L1 300 T > A polymorphism has been demonstrated to disrupt unconventional TMEM59.110-mediated autophagy in mice [484].

### 4.2. Autophagy Activators

#### 4.2.1. Rapamycin

Rapamycin, also known as sirolimus, is a natural mTOR inhibitor [485] that stimulates autophagy both in vitro and in vivo; however, its long-term use is often complicated as it suppresses ribosome biogenesis and protein translation [486]. The rapamycin ester CCI-779/Temsilorimus, which is also an mTOR inhibitor, prevents the development of intracellular tau protein inclusions (abundant protein that stabilizes microtubules in CNS neurons) which are known to accumulate in neurons in Alzheimer’s disease and other tauopathies and can also reduce the density of neurofibrillary tangles by stimulating mTOR-dependent autophagy [487]. RAD001 and AP23573 are rapamycin derivatives with comparatively high safety and low toxicity [488,489]. During the early stages of carotid atherosclerosis, which is an inflammatory step in the primary pathogenesis of cerebrovascular diseases, miRNA-155 plays an important role in the activation of autophagy by rapamycin [490]. Rapamycin has also been shown to increase autophagy, decrease cyclin D1 expression, and attenuate aggressive IgA nephropathy progression in a rat model [491].

#### 4.2.2. Small-Molecule Enhancers of Rapamycin (SMERs)

The immunosuppressive activities of rapamycin limit its frequent use; therefore, safer molecules are required. Three SMERs were identified from 50,729 compounds that can induce autophagy [492]; SMERs 10, 18, and 28 can reduce the pathogenesis associated with polyglutamine aggregation and A53T α-synuclein, which are linked to Huntington’s disease and familial Parkinson’s disease, respectively [493], and induce autophagy in an mTOR-independent manner. In addition, the small molecule AUTEN-99 activates autophagy in cell cultures and animal models and inhibits the progression of neurodegenerative symptoms [343].

#### 4.2.3. Trehalose

Trehalose is a natural disaccharide known to stimulate autophagy via AMPK [494]. In cultured cells from an animal model of Huntington’s disease, trehalose has been shown to stimulate autophagy and reduce Huntingtin protein aggregation during starvation by inhibiting a family of glucose transporters known as the solute carrier 2 or the glucose transporter family [495]. As well as being an autophagy activator and a non-reducing disaccharide, trehalose can also reduce the levels of aggregate-prone proteins and ameliorate cytotoxicity in neurodegenerative disease models [496]. Trehalose treatment has been shown to increase the conversion of LC3-I to LC3-II via an mTOR-independent pathway, whilst sucrose and raffinose are also known to induce autophagy [497]. It has been shown that trehalose can be used to safely induce autophagy in neurodegenerative disorders such as Parkinson’s disease [498], Alzheimer’s disease [499], and prion disease [500]. The bioavailability of trehalose in the body for autophagy induction is low due to the hydrolytic enzyme, trehalase, which is expressed in the intestine and kidney; therefore, the enzyme-stable trehalose analogs, lentztrehaloses A, B, and C, were synthesized and found to be as effective as trehalose [501]. 

#### 4.2.4. Inositol Monophosphatase (IMPase) Inhibitors

Lithium chloride (LiCl) can induce autophagy by inhibiting inositol monophosphatase (IMPase), which reduces inositol and inositol-1,4,5-triphosphate (IP3) levels [502]. Since lithium has already been approved by the FDA for patients with bipolar disorder, it can easily be adapted for other diseases [503]. Long-term oral lithium administration enhanced autophagy in a tauopathy mouse model by inhibiting glycogen synthase kinase-3. L-690,330, a bisphosphonate inhibitor of IMPase, exhibits a similar function to lithium by clearing mutant synucleins and EGFP-HDQ74 [504]. Valproic acid is an angio-suppressive compound which suppresses the Akt/mTOR signaling pathway by acting as a histone deacetylase inhibitor and promotes autophagy, as evidenced by the increased concentrations of LC3-II and Beclin 1 after its administration in prostate cancer cell lines [505]. Carbamazepine and valproic acid can both reduce intracellular inositol-1,4,5 trisphosphate levels [506]. Therefore, other IMPase inhibitors could also be incorporated into therapeutic strategies. 

#### 4.2.5. Epigenetic Changes

Anacardic acid, curcumin, garcinol, and spermidine increase autophagy by reducing the level of acetylation in cultured human cells, as evidenced by depleted sequestosome-1 levels and mTORC1 inhibition [507]. Spermidine also inhibits EP300 and the major autophagy proteins, Atg5, Atg7, Atg12, and LC3 to repress autophagy [508]. Supplementing the diet of mice with coffee beans rich in polyphenols concomitantly increased autophagy and decreased acetylation levels [509], whilst natural polyamines can inhibit acetyltransferases, and their dietary intake can improve the life span of short-living mouse strains and the health of long-living ones [510]. 

#### 4.2.6. Other Molecules

Fluoxetine, which selectively improves the secretion of the pro-inflammatory cytokine TNF-α, and gefitinib, which inhibits the epidermal growth factor receptor (EGFR), can also enhance autophagy to impart neuroprotection and restrict *M. tuberculosis* growth [511]. The antiepileptic compound, carbamazepine, has been found to protect cells against *M. tuberculosis* infection, likely by inducing autophagy [512]. Elevated intracytosolic Ca^2+^ levels can inhibit autophagy; penitrem A, which inhibits Ca^2+^-dependent K^+^ channels, is known to induce autophagy and has been used to treat neurodegenerative disorders [513,514]. Furthermore, bacterial pore-forming toxins can induce the expression of the transcription factor HLH-30 (TFEB) in *C. elegans*, which stimulates autophagy genes and thus inhibits bacterial infection [215]. Metformin, an AMPK activator, has been shown to induce autophagy and reduce the risk of hepatocellular carcinoma (HCC) in diabetic patients [515].

A patent studied by Gorski and Qadir [402] revealed the potential of a thioxanthone-based autophagy inhibitor for suppressing *Atg* gene expression by using siRNA-directed therapy against cancer cells receiving anti-cancer therapies, and for treating cancer in combination with other therapies. The use of dimeric quinacrine derivatives to inhibit lysosomes and autophagy in cancer cells where autophagy allows them to survive metabolic and therapeutic stresses [403] is currently at the patent filing phase. The herbal product Onjisaponin B, from Radix Polygalae (*Polygala tenuifolia*)—a traditional Chinese herb, is known to enhance autophagy. Upon administration, it prevents, treats, or delays the onset of neurodegenerative diseases, thus a patent for this product has been granted to Law et al. [406]. Imidazo [4–5f] [1,10] phenanthroline derivatives (1–6) have been evaluated for their anti-cancer effects, with their use of upregulating LC3-II and Beclin 1 expression and thus inducing autophagy. Of the different compounds evaluated, the phenanthroimidazole 6 derivative was found to induce autophagy and apoptosis; thus, it could prove to be a novel anti-cancer drug [516]. In nanosilica stimulated RAW264.7 cells, melatonin was found to increase LC3 expression and decrease Bax expression, suggesting that autophagy was promoted, and apoptosis was inhibited [517]. Sasanquasaponin ΙΙΙ obtained from *Schima crenata* Korth was found to upregulate LC3-II expression in melanoma cells and induce autophagy; hence, it can be used as an anti-cancer drug to treat melanoma [518]. Epigallocatechin gallate (EGCG) was found to promote autophagosome formation and increase lysosome acidification in Müller cells, thus increasing autophagy. A gliosis experimental model revealed that EGCG reduced reactive gliosis and retinal damage; hence, EGCG should be explored as a treatment of diabetic retinopathy [519].

**Figure 7:**
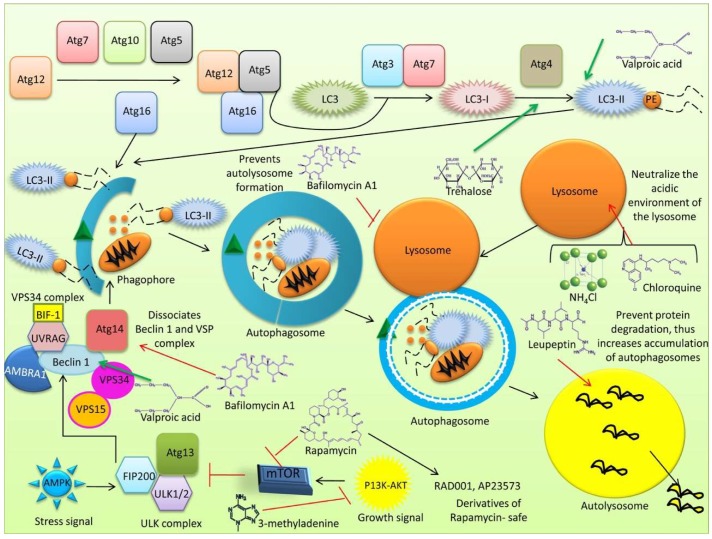
Compounds that inhibit and activate autophagy. Autophagy inhibitors: 3-methyladenine inhibits PI3K. Bafilomycin A1 causes dissociation of the Beclin 1-Vps34 complex and prevents the formation of autolysosome. Chloroquine/hydroxychloroquine, NH_4_Cl, and leupeptin rapidly neutralizing the acidic environment of the lysosome and are used to block lysosomal degradation of substrates. Leupeptin inhibits cysteine, serine and threonine peptidases, and hence blocking protein degradation at the last step of autophagy. Autophagy activators: rapamycin inhibits the mTOR. RAD001 and AP23573 are rapamycin derivatives having comparatively higher safely with minimum dose toxicities. Trehalose causes LC3-I to LC3-II conversion in an mTOR-independent pathway. Valproic acid increases LC3-II and Beclin 1 concentrations.

Compounds that inhibit and activate autophagy are depicted in Figure 7.

## 5. Concluding Remarks and Future Perspectives

Autophagy is a fast-moving area of science which has had an excellent positive impact on animal and human health-related issues and threatening diseases and has considerable future potential. Autophagy is a highly complex process, and understanding the complexity of its mechanisms and internal regulations will be highly beneficial for developing novel methods such as simple ameba-based experimental models. Emerging studies have established many interesting connections in the field of autophagy research. Since autophagy is one of the most conserved evolutionary processes, present in lower organisms and all highly evolved mammals, much work has been carried out to understand its physiological and pathological features. Pathogenesis-associated autophagy is involved in degenerative, inflammatory, infectious, and neoplastic diseases, whereas physiological autophagy is associated with maintaining liver iron homeostasis, CNS development, endothelial cell alignment, atheroprotection, and preventing infection.

Numerous mechanisms of action involving various signaling pathways have been linked to autophagy, with novel mechanisms currently being investigated. mTOR acts as a nutrient sensor in autophagy; however, the process may also be regulated via mTOR-independent pathways. Intracellular Ca^2+^ regulates autophagy, with intracellular Ca^2+^ mobilization via the IP3 receptor stimulating calmodulin and ERK pathways to inhibit autophagy. Notably, Ca^2+^ also inhibits autophagy by increasing mitochondrial ATP levels, which inhibits the CaMKKβ/AMPK pathway, yet promotes autophagy by promoting the AMPK-dependent inhibition of mTOR. Autophagy is linked with other cellular mechanisms such as apoptosis, necrosis, autosis, and necroptosis, and can take the form of microautophagy, macroautophagy, and CMA.

Autophagy can be selective, neutralizing specific substances, or non-selective, degrading different materials irrespective of their nature. It can play physiological and pathological roles by maintaining homeostasis and causing disease, thereby affecting both health and disease. Autophagy encounters infectious and non-infectious diseases, either mediating protection or aggravating the disease. Autophagy has dual roles in an anti-bacterial, anti-viral, and tumor suppressing capacity and by favoring bacterial infections, viral infections, and tumor progression. Among its dual physio-pathological roles, autophagy can promote brain development, immunity, and cardiovascular and endocrine development, or cause neurodegeneration, autoimmune diseases, cardiovascular diseases, obesity, and diabetes.

Autophagy has a genetic basis and many diseases are caused by genetic defects in autophagy genes, including SENDA, Crohn’s disease, HSP, Danon disease, XMEA, and sIBM.

Efforts are being made to explore the positive aspects of autophagy to overcome various health and disease problems. Novel therapeutics and interventions are being investigated to counter autophagy-associated diseases, including apoptosis inhibitors and activators. Excessive autophagy stimulation may cause self-destruction which could be controlled with drugs such as vacuolar-type H (+)-ATPase inhibitors, cycloheximide, lysosome alkalizers (chloroquine, hydroxychloroquine, NH_4_Cl, and neutral red), and acidic protease inhibitors (E64d and pepstatin A), whilst knocking down Beclin 1 and Atg5 using MiR-30a could be used to inhibit the excessive cannibalism caused by autophagy. Upregulating autophagy could therapeutically benefit a range of diseases caused by reduced neuronal apoptosis, including the neurodegeneration-associated impairment of learning/memory capabilities, motor dysfunction, seizures, adult stroke, neonatal asphyxia, cardioskeletal myopathy, and cancers. Autophagy could also prevent various bacterial and viral diseases, inflammatory and autoimmune conditions, and also increase lifespan. Rapamycin, small-molecular enhancers of rapamycin (AUTEN-9), trehalose, IMPase inhibitors (carbamazepine and valproic acid), epigenetic modulators (anacardic acid, curcumin, garcinol, and spermidine), and chemicals (fluoxetine, penitrem A, and metformin) are all autophagy stimulators which help to ameliorate disorders associated with reduced autophagy.

The apparent dual role of autophagy may be due to our poor understanding of this ubiquitous cellular recycling system. Understanding the differences between physiological and pathological autophagy may help us design therapeutic strategies to target pathological autophagy without hindering its physiological effects.

Studies on mouse models combined with human genetic studies provide an important insight into the role of autophagy in neurological diseases like Parkinson’s and inflammatory disorders like Crohn’s disease. Some critical issues have yet to be addressed regarding the role of autophagy in therapeutics and diagnostics. There are few efficient markers for studying autophagy modulation and those that exist have limitations. These markers are important for determining the effect of autophagy on disease initiation and progression. Furthermore, autophagy modulating drugs are imprecise and nonspecific; hence, more specific drugs are required. Similarly, autophagy upregulation appears to be beneficial for removing misfolded and aggregated proteins, intracellular pathogens, damaged mitochondria, and other organelles; however, it is not yet clear how selective autophagy could be upregulated in other situations. These are just some of the questions that need to be addressed in order to use autophagy as a therapeutic molecular process. Nonetheless, autophagy modulation-based therapies will soon become a reality and will help safeguard human health against various pathological conditions.

## Figures and Tables

**Table 1:** Potential role of autophagy in ameliorating/deteriorating diseases and homeostasis.

S. No.	Activity Associated with Autophagy	Effect of Autophagy	Modus Operandi of Related Activity and Example/Proof of Concept	Reference(s)
1	Viral infection	Anti-viral activity	Endogenous viral antigen presentation on MHC class-1 in Herpes simplex virus type 1 (HSV-1) infection	English et al., 2009 [236]
			Delivery of viral antigens to Toll-like receptors (TLRs)- in Vesicular stomatitis virus (VSV) infection; Pattern recognition receptor Toll-7 mediated PI3K-Akt-signaling	Shelly et al., 2009 [239], Nakamoto et al., 2012 [240]
			Sirtuin 1, a NAD(+)-dependent deacetylase mediated dendritic cell and autophagy induction - Respiratory syncytial virus (RSV)	Owczarczyk et al., 2015 [242]
			Autophagy by salicylamide derivates- anti-viral activity against - Cytopathic bovine viral diarrhea virus (cp-BVDV) flavivirus	Needs et al., 2016 [243]
			Inhibition of Sindbis virus replication by overexpression of Beclin 1	Liang et al., 1998 [176]
			Enhanced autophagy by 1α,25-dihydroxycholecalciferol reduces HIV replication	Campbell and Spector, 2012 [220]
			During foot and mouth disease virus infection, Atg5-Atg12 enhances NF-κB and IRF3 pathways	Fan et al., 2017 [244]
			Targeting glycoproteins E1 and E2 and non-structural proteins of Chikungunya virus (CHKV)	Subudhi et al., 2018 [397]
		Pro-viral activity	Rapamycin, chloroquine and small interfering RNAs target Atg5 and Beclin 1- virus production is hampered in New Castle disease virus (NCDV)	Sun et al., 2014 [259]
			Induction of early stages of autophagy and inhibition of later destructive stages – to conquer suppression of new virion production- HIV	Kyei et al., 2009 [261]
			Nef-mediated inhibition of maturation of autophagosome- HIV	
			NS4A-induced autophagy in epithelial cells induces virus replication – Flavivirus	McLean et al., 2011 [269]
			Limitation of autophagosomal by 3-methyladenine or small-interfering RNAs- diminished replication of virus- FMDV	O’Donnell et al., 2011 [272]
			Virus-induced autophagy-mediated impairment of innate immune response- Hepatitis C virus (HCV)	Shrivastava et al., 2011 [252]
			Diminished viral clearance by IFN-α /RBV-based antiviral therapy-HCV	Dash et al., 2016 [274]
			Inhibition of RLR-mediated type-I IFN-independent signaling resulting in antibody-dependent enhancement (ADE) of Dengue virus (DENV)	Huang et al., 2016 [275]
			Adenoviral infection may be privileged by autophagy via an increase in ATP; Atg12-Atg5 complex is significantly upregulated.	Jiang et al., 2008 [277]
			Activation of the phosphatidylinositol 3 kinase/Akt/mTOR pathway and inhibition of autophagy- induce cellular entry of –Human Papilloma virus (HPV) type 16.	Surviladze et al., 2013 [283]
			Replication of Infectious Spleen and Kidney Necrosis virus (ISKNV) is increased when autophagy is induced	Li et al., 2017 [246]
			Human nuclear ribonucleoprotein K (hnRNP-K) and ubiquilin 4 (UBQLN4) help in viral replication. NDP52 human autophagy receptor interacts with CHIKV nsP2 and acts as proviral factor	Wong and Chu, 2018 [37]
			Classical swine fever virus replication is negatively regulated through mTORC1	Luo et al., 2018 [33]
			Autophagosomal targeting of ribosomal proteins by influenza A virus (IAV)	Becker et al., 2018 [32]
			Autophagy of endothelial cells of umbilical vein by Zika virus (ZIKV) helps in replication	Peng et al., 2018 [35]
			Necrosis of cells through severe acute respiratory syndrome-coronavirus (SARS-CoV) open reading frame-3a for multiplication	Yue et al., 2018 [36]
			ER stress by DENV infection helps in autophagy and replication, both in vitro and *in vivo*	Lee et al., 2018 [38]
			Non-structural protein of virus affects mitochondrial membrane in Crimean-Congo Hemorrhagic fever causing apoptosis	Barnwal et al., 2016 [40]
			MDA5 protein inhibition by paramyxovirus V proteins	Mandhana et al., 2018 [41]
			Altering nonstructural proteins of West Nile virus (WNV) affects LC3 modification and aggregation	Martín-Acebes et al., 2015 [43]
2	Bacterial infection	Anti-bacterial activity	In Bcl-xL knockout cells, *Streptococcus pyogenes* infection is promoted	Nakajima et al., 2017 [53]
			NOD proteins interaction with Atg16L1 and initiation of anti-bacterial autophagosome biogenesis	Sorbara et al., 2013 [212]
			Protection from *Caenorhabditis elegans* infection by transcription factor HLH-30/TFEB-mediated autophagy	Chen et al., 2017 [215]
			Inhibition of *Mycobacterium tuberculosis* in human macrophages by SMAD specific E3 ubiquitin protein ligase 1 (SMURF1)	Franco et al., 2017 [398]
		Pro-bacterial activity	Effector Ats-1 is used to enhance autophagosomes formation containing LC3, Beclin 1, Atg8 and Atg6, without lysosomal marker by *Anaplasma phagocytophilum*	Niu et al., 2012 [222]
			*Yersinia-*containing vacuoles (YCVs) contains autophagy markers but not acidified	Moreau et al., 2010 [224]
			*Coxiella*-replicative vacuoles contains LC3, Beclin 1, and Rab24	Vázquez and Colombo, 2010 [226]
			Inside BCVs, replication of *Brucella* requires ULK1, Beclin 1, and Atg14L	Starr et al., 2012 [227]
			Secreted phospholipases C (PLCs; PlcA and PlcB) and a surface protein (ActA) help *Listeria monocytogenes* multiplication	Mitchell et al., 2018 [50]
			*Shigella* gatekeeper protein MxiC regulate type III secretion	Roehrich et al., 2017 [52]
3	Tumor	Tumor suppression	Monoallelic loss of Atg6/Beclin 1 gene – correlated with human prostate, breast, and ovarian cancers	Choi et al., 2013 [285]
			Beclin 1 overexpression inhibits tumor progression	Liang et al., 1999 [286]
			Inhibition of necrosis and chronic inflammation through inhibiting- high mobility group box 1 protein (HMGB1)	Tang et al., 2010 [288]
			Autophagy deficiency- leads to benign hepatoma cell death	Takamura et al., 2011 [291]
			Autophagy induced by PTEN and TSC, the tumor suppressor protein	Feng et al., 2005 [301]; Tsuchihara et al., 2009 [302]
			In mice tumor model, inactivation of Beclin 1 and Atg5 affects autophagy	Levine, 2007 [292]
			Heterozygous disturbance of Beclin 1 lead to development of cancer	Qu et al., 2003 [289]; Yue et al., 2003 [293]
			UV radiation resistance associated gene (UVRAG) can suppress tumorigenicity and proliferation of colon cancer	Liang et al., 2006 [295]
			Pogostone stimulate autophagy and apoptosis through PI3K/Akt/mTOR axis and have anti-colorectal tumor activities	Cao et al., 2017 [303]
		Tumor induction	Autophagy alleviates stressed condition – in hypoxic conditions, metabolic stress, shortage of energy, damaged mitochondria and other organelles	Sato et al., 2007 [306]
			Increased autophagy-associated protein LC3 and BNIP3- linked to colorectal and gastric cancers; Elevated expression of NIP3 (a pro-apoptotic member of the Bcl-2 family of cell death factor) in gastric carcinomas	Lee et al., 2007a [309]
			Autophagy inhibition leads to cell death in tumors acting like an RAS-activated tumor	Guo et al., 2011 [312]
			In the absence of autophagy -accumulation of ubiquitinylated protein aggregates and higher p62 level- responsible for liver tumor	Takamura et al., 2011 [291]
			Activation of autophagy and peroxisome proliferator-activated receptor gamma (PPARγ) protect colon cancer cells against apoptosis	Tylichová et al., 2017 [310]
			In RAS-activated tumors, inhibition of autophagy leads to increased cancer cell death	Guo et al., 2011 [312]
			Post-chemotherapy, increased autophagy may cause cancer cells to go into dormancy and proliferate later	White et al., 2010 [315]
			Proteasome 26S subunit, non-ATPase 10 (PSMD10) or gankyrin induced autophagy in hepatocellular carcinoma causes tumor progression	Luo et al., 2016 [318]
4	Neuronal health	Brain development	Clear protein aggregates / old organelles in old neurons	Hara et al., 2006 [319]
			Atg5 mutation confined to neural tissue leads to impaired growth, progressive motor and behavioral deficits, prominent neurodegeneration and axonal swelling	
			Absence of Atg59 and Atg710- leads to neuronal degeneration	Liao et al., 2007 [100]
			Upon ethanol exposure, autophagy dysregulation in cortical microvessels affects cortical vascular development	Girault et al., 2017 [322]
		Neurodegeneration	Dysregulated autophagy results in accumulation of damaged and toxic molecules- leads to Alzheimer’s, Parkinson’s and Huntington’s diseases	Sahni et al., 2014 [327]
			Anomalies in endosomal-lysosomal pathway and accumulation of autophagosomes- lead to Alzheimer’s, Parkinson’s and Huntington’s diseases	Pickford et al., 2008 [330]
			Beclin 1 deficiency- leads to deposition of β-amyloid protein and neurodegeneration	
			Atg7 mutation in mice causes accumulate ubiquitin and results in neurodegeneration and death	Komatsu et al., 2006 [334]; Nixon, 2013 [31]
			Embryos of Ambra1-deficient mice possess defects in the neuronal tube	Fimia et al., 2007 [335]
			Mutations in the phosphatase and tensin homolog (PTEN)-induced kinase 1 (PINK1) and Parkin genes result in defective mitophagy which leads to Parkinson’s disease	Whitworth and Pallanck, 2017 [339]
			Beta-propeller protein causes neurodegeneration	Stige et al., 2018 [399]
5	Iron availability in body	Homeostasis	Iron in the form of ferritin complex- redox-active iron is sequestered in lysosome	Kurz et al., 2011 [382]; Krishan et al., 2015 [384]
			Knockdown of nuclear receptor co-activator 4 (NCOA4), which is responsible for directing ferritin to autophagosome, increases iron-responsive element-binding protein 2 (IRP2)- prevent cell death by exogenous reactive oxygen species	Berndt, 2014 [386]
		Ferritinophagy	Iron storage protein called ferritin is degraded in the lysosome; thus, resulting in a form of selective macroautophagy	Hamaï and Mehrpour, 2017 [379]
6	Chronic inflammatory bowel disease	Anti-effect	Reduction in TNF-α induced apoptosis in gut epithelium	Pott and Maloy, 2018 [400]
		Pro-effect	Goblet cell function, cytokine production or NOD2, ATG16L1, and IRGM gene regulation affect pathogenesis of inflammatory bowel disease	Iida et al., 2017 [401]
7	Lifestyle diseases	Obesity	Causes biochemical disturbance, ER stress, mitochondrial dysfunction induces obesity-cardiac disorders	Che et al., 2018 [388]
		Diabetes mellitus	Affects beta-cells of pancreas, insulin target tissues, glucose metabolism	Bhattacharya et al., 2018 [389]
		Cardiovascular disease	Perturbations in autophagic machinery in cardiomyocytes and other cardiovascular cell types	Schiattarella and Hill, 2015 [377]
			Autophagy through PARP1 modulation of FoxO3a transcription in cardiomyocytes	Wang et al., 2018a [378]

**Table 2:** Details of applied/granted patents for treating ailments related to autophagy dysfunction.

S. No.	Targeted Ailment	Title of Patent	Patent Number	Modus Operandi	Inventers	Date of Publication	Status	Reference(s)
1.	Tumor treatment	Inhibition of autophagy genes in cancer chemotherapy	US 8076308	Compositions comprise a siRNA directed against an Atg gene to inhibit its expression	Gorski SM, Qadir MA	13.12.11	Granted	Gorski and Qadir 2011 [402]
		Dimeric quinacrine derivatives as autophagy inhibitors for cancer therapy	WO 2016168721	Chloroquine compounds and derivatives mediated inhibition of lysosome	Amaravadi RK, Winkler J.	20.10.16	Application	Amaravadi and Winkler, 2016 [403]
		Anti-cervical cancer compound and method of use thereof	US 9339488	Griffipavixanthone, a novel cytotoxic Bixanthone from *Garcinia griffithii* and *G. pavifolia* selectively kill cervical cancer cells via inducing autophagy	XU H, Zhang H, Lao Y, Wang X, Chen K, Yang D, Chen S, Lin C, Bian Z, Lu A, Chan ASC,	17.05.16	Grant	Xu et al., 2016 [404]
		Substituted thioxanthenone autophagy inhibitors	US 9926326	Inhibition of autophagy through autophagy inhibitors developed from substitution of chemical groups, can help in treatment of cancers	Carew J, Phillips JG	27.03.18	Grant	Carew and Phillips, 2018 [405]
		Method for inhibiting growth of ovarian cancer cells	US20180050012	Method of inhibition of ovarian cancer cells by 4-acetyl-antroquinonol B or its salt	Huang CC, Tzeng YM, Yeh CT, Wu THA	22.02.18	Application	Huang et al., 2018 [25]
2.	Neuroprotection	Autophagy enhancer for treatment of neurodegenerative diseases	US 9005677	Onjisaponin B derived and isolated from *Radix polygalae* enhances autophagy	Law YK, Wu AG, Wong KW, Liu L	14.04.15	Grant	Law et al., 2015 [406]
		Autophagy enhancing compounds, peptides and peptidomimetic compounds for use in the treatment of neuronal diseases	WO 2012076555	Pharmaceutical compositions enhancing autophagy in acute focal brain lesions	D’amelio M, Molinari M, Viscomi MT, Cecconi F	14.06.12	Application	D’amelio et al., 2012 [407]
		Highly potent peptides to control cancer and neurodegenerative diseases	WO 2010011952	Inhibition of autophagy by administering a FLIP protein interfering with the formation of the LC3-Atg4-Atg7-Atg3 conjugation complex	Jung JU, Lee JS	24.06.10	Application	Jung and Lee, 2010 [408]
		Methods for reducing neurodegeneration	EP 2717695	Inhibiting the expression of mTOR in canine	Middleton RP, Zanghi BM	02.11.16	Grant	Middleton and Zanghi, 2016 [409]
		Regulating autophagy	WO2008122038A1	Regulating autophagy helps in prevention and treatment of neurodeneration or other diseases	Bradner JE , Shen JP, Perlstein EO, Rubinsztein D, Sarkar S, Wood SLS	09.10.08	Application	Bradner et al., 2008 [410]
		Method for modulating autophagy and applications thereof	WO2017098467A1	Autophagy modulators (pyridines; hydrogenated derivatives) regulate all types of autophagy by increasing or decreasing autophagic flux	Manjithaya R, Mishra P, Santhi Natesan S, Bats S, Ammanathan V, Chavalmane A	15.06.17	Application	Manjithaya et al., 2017 [411]
		mTOR-independent activator of TFEB for autophagy enhancement and uses thereof	US 9351946	Small molecules enhance autophagy and lysosome biogenesis by activating the gene TFEB	Li M, Song J, Zeng Y, Liu L	31.05.16	grant	Li et al., 2016 [412]
		Combination product with autophagy modulator	WO 2016131945	Autophagy modulator directly or indirectly acting on a complex involved in autophagy such as ULKl/2-Atgl3- FIP200 complex, Atg9 complex, STING complex, class III PI3K complex, ubiquitin-like conjugation systems Atg5-Atgl2, LC3, fusion complex, SNARE protein and transcription factor EB	Zaupa C, Hortelano J, Silvestre N, Spindler A	25.08.16	Application	Zaupa et al., 2016 [413]
3.	Viral inhibitor	Treatment of hepatitis C virus-related diseases using hydroxychloroquine or a combination of hydroxychloroquine and an anti-viral agent	US 8987302	Chloroquine cause pH-dependent inhibition of degradation of cargo delivered to the lysosome	Halfon P	24.03.15	Grant	Halfon, 2015 [414]
		Enhancing the anti-tumor, anti-viral, and anti-protozoan effects of 2-amino-n-[4-[5-phenanthren-2-yl-3-(trifluoromethyl)pyrazol-1-yl] phenyl]acetamide (osu-03012) and other pharmaceutical drugs	WO 2016069854	Drug OSU-03012 (AR-12) in combination with multikinase inhibitor	Dent P, Zukiwski A, Proniuk S	06.05.16	Application	Dent et al., 2016 [415]
		Autophagy-inducing peptide	CA2864145C	Autophagy-inducing peptide derived from beclin-1 (residues 269-283) has antiviral role against West Nile Virus, chikungunya virus, HIV and Ebola viru	Levine BC, Sanae Shoji-Kawata S, Lichtarge O, Wilkins AD	14.02.17	Grant	Levine et al., 2017 [416]
		Combination treatment of RAS-positive diseases with PDE-delta inhibitor and direct autophagy inhibitor	US 9861623B1	Potentiating the apoptotic activity of deltarasin, a PDE-delta inhibitor by 3-methyladenine, a direct autophagy inhibitor for treatment of RAS positive cases	Liu L, Ward D, Leung ELH, Yao XJ, Wong VKW, Luo LX	09.01.18	Grant	Liu et al., 2018 [417]
4.	Iron homeostasis	Compositions and methods for modulating nuclear receptor coactivator 4 (NCOA4) -mediated autophagic targeting of ferritin	WO 2015149006	Modulation of the level and activity of nuclear receptor coactivator 4 (NCOA4)	Kimmelman AC, Mancias JD, Harper JW	26.11.15	Application	Kimmelman et al., 2015 [418]
		MicroRNA that regulate autophagy	JPWO 2015037656A1	miRNA-mediated targeting the WDR45 gene and ATP13A2 gene	Hidenao S, Jun U, Koichi W	02.03.15	Application	Hidenao et al., 2015 [419]
		Use of hepcidin as a regulator of iron homeostasis	US7169758B2	Hepcidin, a key regulator of the entry of iron into the circulation can be used for disorders of iron overload	Nicolas G, Vaulont S, Kahn A	30.01.07	Grant	Nicolas et al., 2007 [420]
		Erythroferrone and erfe polypeptides and methods of regulating iron metabolism	CA2890040A1	Hepcidin concentration can be regulated by herein polypeptides called as erythroferrone and erfe polypeptides	Ganz T, Nemeth E, Kautz L	08.05.14	Application	Ganz et al., 2014 [421]

**Table 3:** Diseases due to genetic defects in autophagy genes.

S. No.	Name of disorder	Type of disorder	Mutant gene	Outcome of pathological condition	Symptoms	Reference(s)
1	Static encephalopathy of childhood with neurodegeneration in adulthood (SENDA)	Neurodegenerative disorder	*WIPI4* located at Xp11.23	*WIPI4*, homologous to yeast Atg18, is recruited to autophagosome formation site, severely reduced in affected individuals. Iron accumulation in brain	In childhood- early-onset of spastic paraplegia and mental retardation	Ozawa et al. 2014 [445]; Haack et al., 2012 [423]; Saitsu et al., 2013 [424]
					In adult age- symptoms of parkinsonism and dystonia	
2	Crohn’s disease	Inflammatory bowel disease	*Atg16L1*	Inhibited LC3 conjugations to phosphatidylethanolamine (PE)	Abdominal pain, diarrhea, vomiting, and weight loss	Fujita et al., 2009 [430]
3	Hereditary spastic paraparesis (HSP)	Increased muscle spasticity	A recessive mutation in *TECPR2*	Autophagosome accumulation due to impaired fusion with lysosome	Lower extremity weakness and spasticity	Vantaggiato et al., 2013 [433]; Oz-Levi et al., 2012 [432]
4	Danon disease	Cardiomyopathy and intellectual dysfunction	Lysosome-associated membrane protein 2 (LAMP-2B isoform)	Accumulation of autophagic vacuoles in liver, kidney, pancreas, and cardiac and skeletal muscles	Weakening of skeletal muscles	D’souza et al., 2014 [446]; Rothaug et al., 2015 [435]; Ng et al., 2016 [436]
5	X-linked myopathy with excessive autophagy (XMEA)	Skeletal myopathy	*VMA21*	Elevated levels of CPK	Weakness in proximal muscles of the legs	Ruggieri et al., 2015 [440]; Dowling et al., 2015 [439]
				Interrupted sarcolemma membrane homeostasis Impaired autophagy and lysosomal function Vps15 knockouts exhibit muscle pathology similar to the XMEA, indicative of role of aberrant autophagy in XMEA disease.		
6	Sporadic inclusion body myositis (sIBM)	Progressive muscle disorder	MYH2	Muscle tissues exhibits both inflammatory and degenerative changes. Impaired autophagy with inhibited lysosomal protein degradation.	Progressive quadriceps femoris and deep finger flexors weakness and atrophy	Mastaglia, 2009 [444]; Nogalska et al., 2010 [442]
7	Vici syndrome	Callosal agenesis, cataracts, hypopigmentation, cardiomyopathy, psychomotor retardation, and immunodeficiency with cleft lip and palate	Recessive mutations in EPG5	Deficiency blocks the maturation of autophagosomes into degradative autolysosomes resulting in accumulation of non-degradative autolysosomes.	Psychomotor abnormalities	Cullup et al., 2013 [447]
				Defect in the endocytic pathway Epg5 deficiency blocks the maturation of autophagosomes into degradative autolysosomes	Facial dysmorphism and cataracts	Zhao et al., 2013 [448]
8	Lysosomal storage disorder Niemann-Pick type C (NPC)	Neurodegeneration and liver dysfunction	NPC1 or NPC2	Compromised autophagy with accumulation of autophagosomes as evidenced by elevated LC3-II levels and LC3 positive vesicles in the NPC1 mutant cells Cholesterol accumulation	Increased death of brain and liver cells	Sarkar et al., 2013 [449]
9	Pompe disease	Pathology of the neuromuscular junction	Lysosomal acid α-glucosidase	Accumulation of glycogen in the nervous system	Muscle atrophy, weakness, loss of muscle function and cardio-respiratory failure	Todd et al., 2015 [450]
10	Cancer	High percentage of human breast cancers and ovarian cancer	Monoallelic deletion of *BECN1*	Activation of inflammasome leading to the maturation of inflammatory cytokines like IL-1β and IL-18	Cell necrosis, chronic inflammation and ultimate tumorigenesis	Sun et al., 2013 [451]
11	Myelodysplastic syndromes / acute myeloid leukemia (AML)	Accumulation of damaged mitochondria	Atg7	Increased levels of ROS	Bone marrow cells are characterized by mitochondrial abnormalities and increased cell death	Watson et al., 2011 [452]
12	Autoimmune/Autoinflammatory diseases	Immune dysfunction	Atg5	Disturbance in autophagic vesicle formation, immune cell development and function, mitochondrial ROS, antimicrobial immunity [(retinoic acid receptor responder 3 (RARRES3) and mitochondrial anti-viral signalling protein (MAVS)]	Autoimmune diseases, susceptibility to infections, e.g., HIV, bacteria	Ye et al., 2018 [453]
13	Autoimmune thyroid diseases	Autoimmune thyroid diseases and Graves’ disease	IRGM	Lymphocytic infiltration in thyroid, antibodies to antigens including thyroid-stimulating hormone receptor (TSHR), thyroid antigens including thyroglobulin (Tg), and thyroid peroxidase (TPO), anti-TSHR autoantibodies	Graves’ disease, hyperthyroidism	Yao et al., 2018 [454]
14	Paget disease of bone (PDB)	Osteoclast size, number and activity increase causing bone cleavage, cytoplasmic inclusions with protein aggregates responsible for autophagy	Atg2B, Atg5	Focal bone disorder affecting the skeleton segmentally, bone defects, softening, breakage	Bone defects, softening, breakage, pain	Usategui-Martín et al., 2015 [455]
15	Celiac disease in children	Chronic systemic autoimmune disease of the small intestine, gluten induced damage resulting in celiac sprue and gluten-sensitive enteropathy	Atg7 and Beclin 1	Chronic systemic autoimmune disorder, enteropathy	Diarrhea, dehydration, indigestion, decreased appetite, stomach-ache and bloating, poor growth, and weight loss	Comincini et al., 2017 [456]
16	Huntington disease	Neurodegenerative disorder, CAG trinucleotide repeats in the 5′ coding region of the IT15 (Interesting Transcript 15) gene of 4th chromosome	IT15	Neurodegeneration, deformity in autosomal-lysosome degradation, neurodegenerative proteinopathy, accumulation of toxic materials	Depression, apathy, irritability, suicidal behaviours, involuntary movements/chorea, progressive dementia, severe weight loss	Barboza and Ghisi, 2018 [457]
17	Cardiac disease	Cardiac autophagy disorder	Beclin 1, microRNAs (miRNAs)	Over or under expression of genes regulating cardiac autophagy (Beclin 1), miRNAs regulate cardiac autophagy by suppressing the expression of autophagy-related genes in a targeted manner	Signs of cardiovascular disease- like heart attack, pain, fainting, dysrhythmia	Shirakabe et al., 2016 [458]; Sun et al., 2018 [459]
